# Interindividual Variation in Gut Nitrergic Neuron Density Is Regulated By GDNF Levels and ETV1

**DOI:** 10.1016/j.jcmgh.2024.101405

**Published:** 2024-09-17

**Authors:** Heikki T. Virtanen, Peyman Choopanian, L. Lauriina Porokuokka, Richard Forsgård, Daniel R. Garton, Soophie Olfat, Riitta Korpela, Mehdi Mirzaie, Jaan-Olle Andressoo

**Affiliations:** 1 Translational Neuroscience, Department of Pharmacology, Faculty of Medicine and Helsinki Institute of Life Science, University of Helsinki, Helsinki, Finland; 2 Department of Pharmacology, Faculty of Medicine, University of Helsinki, Helsinki, Finland; 3 Division of Neurogeriatrics, Department of Neurobiology, Care Sciences and Society (NVS), Karolinska Institutet, Huddinge, Sweden

**Keywords:** Endogenous GDNF, MicroRNAs, ETV1, NOS1, Nitrergic Neurons, Gut Function

## Abstract

**Background & Aims:**

The size and function of the enteric nervous system (ENS) can vary substantially between individuals. Because ENS function is involved in the etiology of a growing number of common human diseases, understanding mechanisms that regulate ENS variation is important.

**Methods:**

We analyzed RNAseq data from 41 normal adult human colon biopsies and single-cell RNA-seq data from human and mouse developing gut. To establish cause-consequence relationship we used alleles in mice that allow levels change of the candidate effector molecule in the comparable range to human samples. We used siRNA and primary neuronal cultures to define downstream molecular events and characterized gut functional changes in mice where molecular phenotypes paralleled findings in humans.

**Results:**

We found that glial cell line–derived neurotrophic factor (GDNF) levels in the human colon vary about 5-fold and correlate strongly with nitrergic marker expression. In mice, we defined that GDNF levels are regulated via its 3’ untranslated region (3’ UTR) in the gastrointestinal tract and observed similar correlation between GDNF levels and nitrergic lineage development. We identified miR-9 and miR-133 as evolutionarily conserved candidates for negative regulation of GDNF expression in the gastrointestinal tract. Functionally, an increase in inhibitory nitrergic innervation results in an increase in gastrointestinal tract transit time, stool size, and water content accompanied with modestly reduced epithelial barrier function. Mechanistically, we found that GDNF levels regulate nitrergic lineage development via induction of transcription factor ETV1, corroborated by single-cell gene expression data in human and mouse developing enteric neurons.

**Conclusions:**

Our results reveal how normal variation in GDNF levels influence ENS size, composition, and gut function, suggesting a mechanism for well-known interindividual variation among those parameters.

**Summary**

Our work shows that some interindividual differences in the enteric nervous system composition and hence gastrointestinal tract function likely relate to normal interindividual variation in the levels of glial cell line–derived neurotrophic factor, a protein known to regulate enteric nervous system development.

It has become clear that enteric nervous system (ENS) size and composition display a remarkable at least 2- to 3-fold, variation between individuals.^1^, ^2^, ^3^, ^4^, ^5^, ^6^ Recent research has revealed that ENS size and function is implicated in the cause of the increasing number of human diseases from irritable bowel syndrome^7^ and neurologic disorders^7^ to cancer.^7^^,^^8^ Therefore, understanding mechanisms that regulate normal ENS variation is critically important, because it would provide knowledge base for linking normal variation to specific disease susceptibility and progression in the future.

Glial cell line–derived neurotrophic factor (GDNF) is an essential ENS morphogen with temporally divided pleiotropic effects in the ENS development. GDNF first acts as a chemoattractant for directed migration of enteric neural crest–derived cells^9^^,^^10^ in addition to regulating enteric neural crest–derived cell survival,^11^ proliferation,^9^^,^^11^ and differentiation at the later stages of ENS development.^12^ Because of its temporally divided pleiotropic effects, analysis of GDNF level effects by ectopic GDNF expression may be challenging. An ability to regulate endogenous GDNF expression in the range of normal variation, however, has not been possible. Thus, how much GDNF levels normally vary between individuals, how endogenous GDNF levels are regulated, and how this influences ENS size, composition, and function has remained unaddressed.

Here we analyzed RNAseq data from 41 normal adult human colon biopsies (http://gepia2.cancer-pku.cn/) and single-cell RNA-seq data from human and mouse developing gut. To establish cause-consequence relationship we used unique alleles in mice that allow change of endogenous candidate effector molecule (GDNF) levels in the comparable range to human samples. We used small interfering RNA (siRNA) and primary neuronal cultures to define downstream molecular events and characterized gut functional changes in mice where molecular phenotypes paralleled findings in humans.

Our results reveal how variation in GDNF levels influence ENS size, composition, and gut function, suggesting a mechanism for well-known interindividual variation in those parameters.

## Results

### GDNF Levels Vary 5-Fold in Adult Human Colon and Positively Correlate with Pan-neuronal Markers UCHL1 and ELAVL4 and Nitrergic Marker Nitric Oxide Synthase Levels

**Figure 1:**
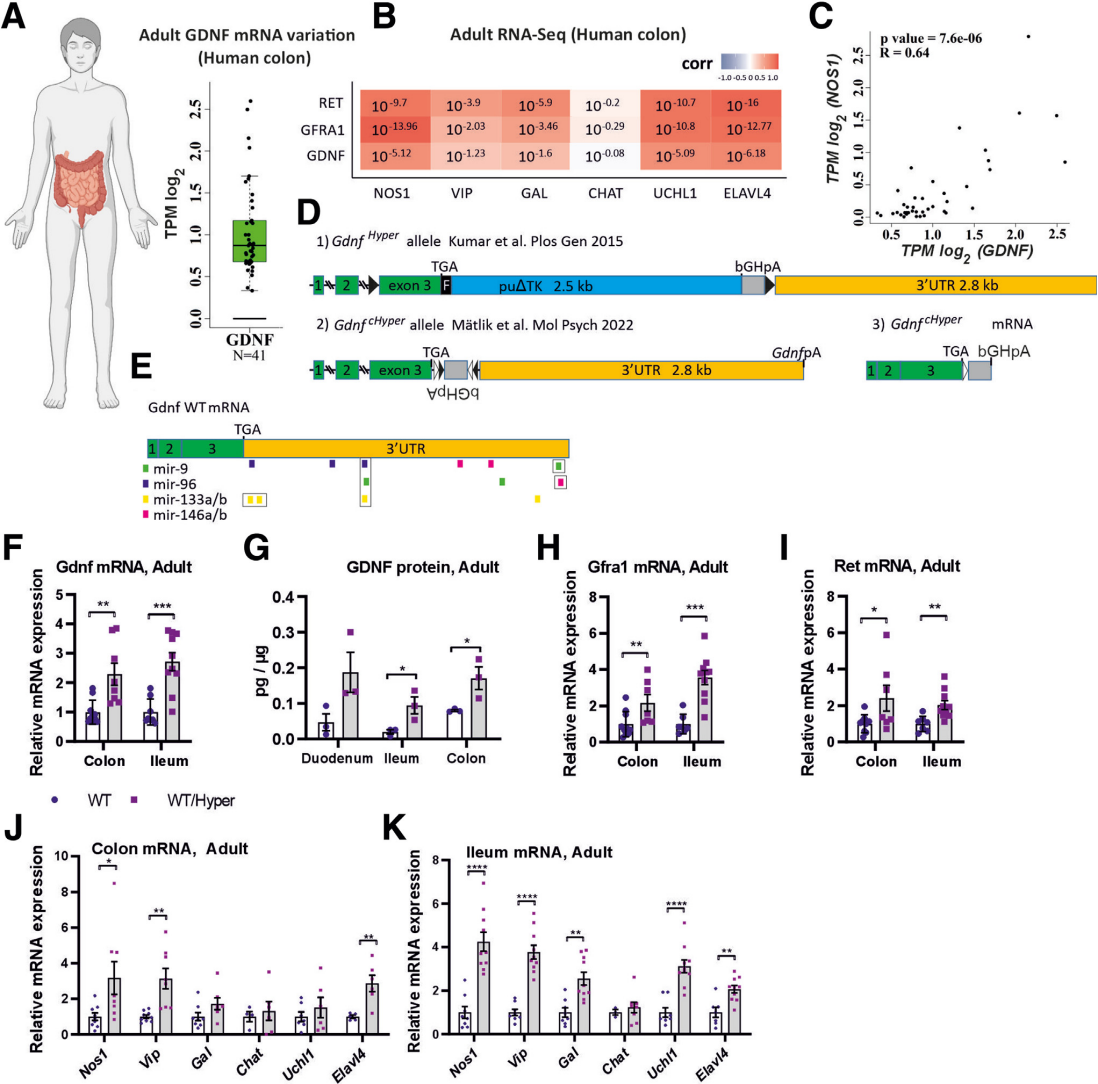
**Analysis of GDNF and ENS markers expression in human and mouse GI tracts.** (*A*) Variation in GDNF transcript levels in adult human colon samples derived from the GEPIA online tool based on the TCGA database (image created using biorender.com). The y-axis represents the TPM (transcript per million) values in log2 scale. Min-max range in non log2 scale 1.23–6.06 TPM. n = 41 healthy human colon samples were analyzed (box plot representation in which the *whisker* denotes 95% confidence interval, the *boxes* indicate the first and third quartiles, and the *line* within the boxes is median value). (*B*) Pairwise comparison of GDNF or GFRA1 or RET with common enteric neuronal markers. The Pearson correlation was used to determine a linear relationship. Values smaller than 10^-1.3^ (*P* < .05) indicate significance. (*C*) Scatter plot showing the relationship between GDNF TPM values and NOS1 TPM values in log2 scale. The data were acquired from GEPIA2-database (Gene Expression Profiling Interactive Analysis). (*D*) Alleles for 2 different mouse models with *Gdnf* 3’UTR replacement: (1) *Gdnf* hypermorphic allele^14^ where the native *Gdnf* 3’UTR is replaced with a puroΔtk cassette, which lacks the microRNA (*E*, *colored bars*) binding sites. (2) *Gdnf*^*cHyper*^ allele containing the bGHpA in an inverted orientation flanked by the FLEx system.^15^ Exons and the 3’UTR are drawn to scale. (*E*) *Gdnf* WT allele, and the resulting mRNA with predicted binding sites for miR-9, miR-96, miR-133a/b, and miR-146a/b. (*F*) In adult *Gdnf*^*WT/Hyper*^ mice (adult, defined here and elsewhere as 2–6 months of age) mRNA levels (qPCR analysis) of Gdnf were increased both in colon (n = 8–10 mice per genotype; ∗∗*P* < .01) and ileum (n = 8–10; ∗∗∗*P* < .001). (*G*) GDNF protein (enzyme-linked immunosorbent assay analysis) levels are increased in the ileum (n = 3; ∗*P* < .05) and colon (n = 3; ∗*P* < .05). (*H*) mRNA levels (qPCR analysis) of Gfra1 were increased both in the colon (n = 7–10; ∗∗*P* < .01) and the ileum (n = 7–10; ∗∗∗*P* < .001). (*I*) mRNA levels (qPCR analysis) of Ret were increased both in the colon (n = 7–9; ∗*P* < .05) and the ileum (n = 7–10; ∗∗*P* < .01). (*J*) qPCR analysis of mRNA levels of neuronal markers in the colon revealed upregulation of Nos1, Vip, and Elavl4 (n = 4–9; ∗*P* < .05; ∗∗*P* < .01). (*K*) qPCR analysis of mRNA levels of neuronal markers in the ileum revealed upregulation of Nos1, Vip, Gal, Uchl1, and Elavl4 (n = 3–10; ∗∗*P* < .01; ∗∗∗∗*P* < .0001).

To understand how GDNF levels vary in human gut samples we reanalyzed an open source RNAseq dataset from healthy adult human colon biopsies (http://gepia2.cancer-pku.cn/).^13^ Analysis of GDNF mRNA revealed several-fold variation in GDNF levels between individuals across 41 human samples (Figure 1*A*). Next, we analyzed whether GDNF levels correlate with common enteric neuronal markers. We found that in the human colon, there is a strong positive correlation between GDNF levels and the levels of pan-neuronal genes UCHL1 and ELAVL4 (Figure 1*B*). Moreover, we observed a strong positive correlation between GDNF and nitric oxide synthase (NOS1) (Figure 1*B*, *C*) and a correlation between GDNF and GAL and VIP, 2 genes that are coexpressed in nitrergic neurons (Figure 1*B*). No correlation between GDNF and CHAT, the main marker of cholinergic neurons, was observed (Figure 1*B*).

### Altering GDNF Expression Levels in Mice Yields in Similar Correlation in ENS Marker Expression

We have previously demonstrated that replacement or conditional replacement of GDNF 3ʹUTR in mice (Figure 1*D*) with a 3ʹUTR with reduced responsiveness to negative regulators, such as microRNAs miR-9, miR-96, miR-133b, and miR-146 (Figure 1*E*), increases endogenous GDNF by approximately 2- to 4-fold at the posttranscriptional level in the brain, spinal cord, kidney, and testis.^14^^,^^15^ The Gdnf alleles were designated *Gdnf*^*Hyper*^^14^ and *Gdnf*^*cHyper*^^15^ to denote constitutive or conditional hypermorph alleles, respectively. First, we investigated how GDNF levels are affected in adult guts of *Gdnf* ^*WT/Hyper*^ mice to assess whether these mice could be used to model the natural variation in GDNF expression levels observed in the human gut. We observed a 2- to 3-fold increase in Gdnf mRNA in *Gdnf* ^*WT/Hyper*^ mice compared with wild-type (WT) mice (Figure 1*F*). GDNF protein analysis by enzyme-linked immunosorbent assay in the duodenum, ileum, and colon revealed that protein levels are also increased by several folds in *Gdnf* ^*WT/Hyper*^ mice compared with WT mice (Figure 1*G*). Additionally, the mRNA levels of the GDNF coreceptor Gfra1 and receptor Ret*,* to which neuronal marker levels in the human dataset also correlate (Figure 1*B*), are upregulated in adult mouse gut (Figure 1*H*, *I*).

Next, we were interested to see whether the expression of common ENS markers in *Gdnf* ^*WT/Hyper*^ mice was comparable with the human results. The expression pattern of ENS markers in *Gdnf* ^*WT/Hyper*^ mice mirrored the human results both in the colon (Figure 1*J*) and in the ileum (Figure 1*K*), in that Gdnf and Gfra1 mRNA levels correlated strongly with nitrergic marker expression.

**Figure 2:**
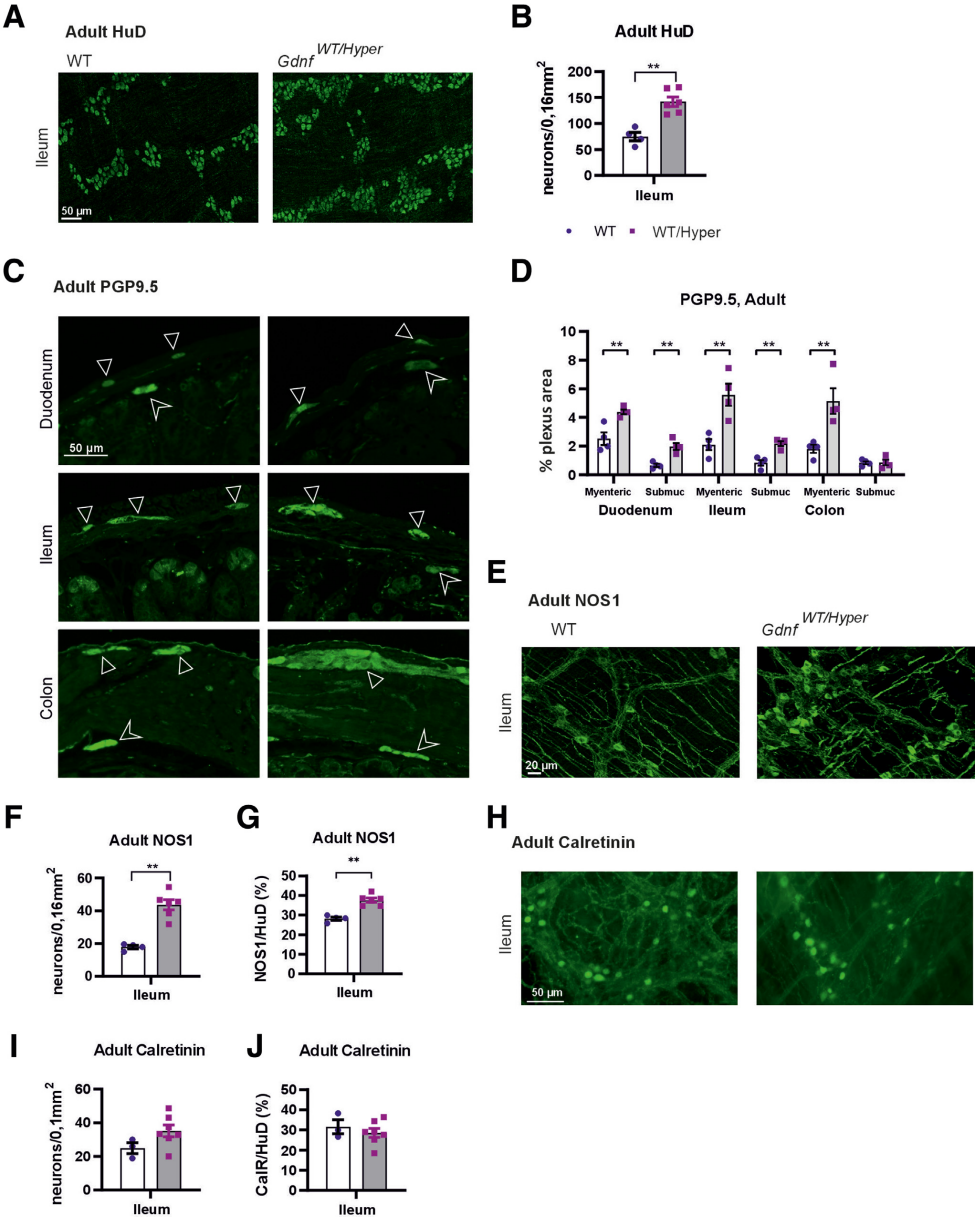
**Analysis of 3’UTR controlled GDNF levels effect on ENS size and composition.** (*A*) Representative images of adult whole mount LMMP preparations of the ileum stained with anti-HuD for enteric neurons. (*B*) The number of enteric neurons in the ileum in *Gdnf*^*WT/Hyper*^ mice compared with WT littermates is increased (n = 4–6; ∗∗*P* < .01). (*C*, *D*) Increase in PGP9.5 positive area in adult *Gdnf*^*WT/Hyper*^ mice except for the submucosal plexus of the colon. (*C*) Representative images. (*D*) quantification (n = 4; ∗∗*P* < .01). (*E*) Representative images of adult whole mount LMMP preparations of the ileum stained with anti-NOS1 for nitrergic neurons. (*F*) The number of nitrergic neurons in *Gdnf*^*WT/Hyper*^ mice is increased compared with WT littermates in the ileum (n = 4–6; ∗∗*P* < .01). (*G*) Relative number of nitrergic neurons compared with non-nitrergic neurons in *Gdnf*^*WT/*^^*H*^^*yper*^ mice is increased compared with WT littermates in the ileum (n = 4–6; ∗∗*P* < .01). (*H-J*) In adult *Gdnf*^*WT/Hyper*^ mice no increase in the number of calretinin-positive neurons is observed. (*H*) Representative images, (*I*, *J*) quantification (n = 3–7).

Analysis of enteric neurons in the adult mouse (defined as 2–6 months of age) gut using an antibody against the pan-neuronal marker HuD (ELAVL4) revealed that *Gdnf* ^*WT/Hyper*^ mice have approximately 2 times more enteric neurons in the gut (Figure 2*A*, *B*). In addition, the area covered by PGP9.5 was increased by about 2-fold in the myenteric plexus, in line with neuronal number counts, and by approximately 3-fold in the submucosal plexus apart from in the colonic submucosal plexus (Figure 2*C*, *D*). Immunohistochemical analysis of nitrergic neurons revealed that GDNF upregulation via abolishing inhibition of GDNF expression through its native 3’UTR resulted in a 2.5-fold increase in the number of nitrergic neurons (Figure 2*E*, *F*), which corresponded to 30% of relative increase in the number of nitrergic neurons compared with nonnitrergic neurons (Figure 2*G*). No statistically significant increase in number of calretinin-positive neurons was observed (Figure 2*H-J*).

### miR-9 and miR-133b Are Candidate Negative Regulators of GDNF Levels in the Developing Mouse Gut

**Figure 3:**
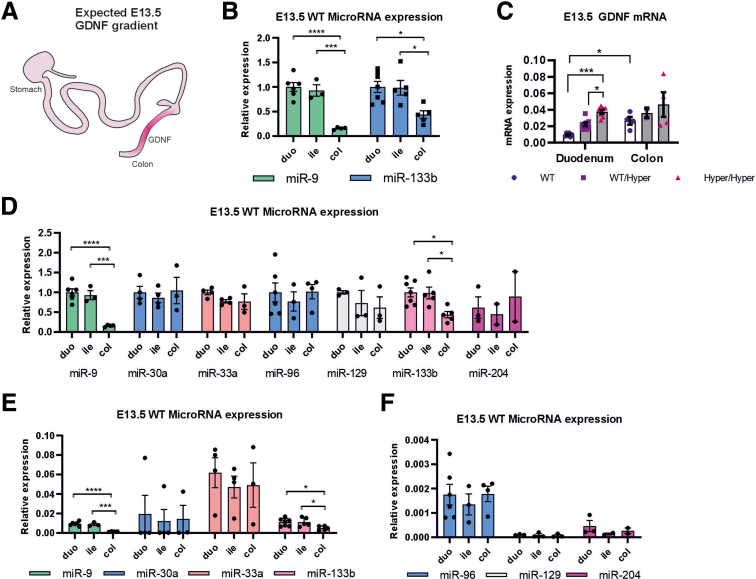
**Analysis of GDNF-regulating microRNAs and Gdnf expression gradient in the developing gut.** (*A*) GDNF is a strong chemoattractant for enteric neural crest–derived cells during colonization of the gut by enteric nervous system precursors. At E13.5 colonization is nearly complete and GDNF gradient expression is expected to be highest in the colon. (*B*) miR-9 and miR-133b expression relative to reference RNA snoRNA202 in WT developing gut at E13.5 is significantly different in duodenum (duo) and ileum (ile) versus colon (col); miR-9 duo (n = 6) versus col (n = 4) ∗∗∗∗*P* < .0001, ile (n = 3) versus col (n = 4) ∗∗∗*P* < .001; miR-133b duo (n = 7) versus col (n = 5) ∗*P* < .05, ile (n = 5) versus col (n = 5) ∗*P* < .05. MiRNA expression levels are normalized to duodenum average expression (set as 1). (*C*) Gdnf mRNA expression in WT mice is higher (∗*P* < .05) in the colon (n = 4) compared with the duodenum (n = 6) and inversely correlates with miR-9 and miR-133b expression. In the duodenum, *Gdnf*^*Hyper/Hyper*^ mice have increased GDNF levels compared with *Gdnf*^*WT/Hyper*^ and WT mice (n = 6–7; ∗*P* < .05; ∗∗∗*P* < .001). In the colon, GDNF levels are not altered between the genotypes (*Gdnf*^*Hyper/Hyper*^ and WT mice n = 4; *Gdnf*^*WT/Hyper*^ mice n = 2). (*D*) microRNA relative expression in the gut with expression in duodenum set as 1s. (*E*, *F*) Expression levels of studied microRNAs normalized to snoRNA202. The microRNAs are grouped based on their abundance for better representation. miR-9 duo (n = 6) versus col (n = 4) ∗∗∗∗*P* < .0001, ile (n = 3) versus col (n = 4) ∗∗∗*P* < .001; miR-133b duo (n = 7) versus col (n = 5) ∗*P* < .05, ile (n = 5) versus col (n = 5) ∗*P* < .05.

Previously, we validated functional binding sites for miR-9 and miR-96 in the *Gdnf* 3’UTR and identified binding sites for miR-133b in the *Gdnf* 3’UTR (Figure 1*E*).^14^ We analyzed the above microRNAs, the expression of other in silico predicted Gdnf mRNA interacting microRNAs, and Gdnf mRNA levels in the WT gut at E13.5 when GDNF levels are known to be high in the caecum and colon (Figure 3*A*), thereby guiding the enteric neural crest–derived cell migration and proliferation in this gut region.^10^ We found that in the WT gut, miR-9 and miR-133b are relatively abundant in the small intestine and low in the colon at that age (Figure 3*B*) matching low Gdnf mRNA levels in the duodenum and high Gdnf mRNA levels in the colon (Figure 3*C*). In the duodenum where expression levels of miR-9 and miR-133b are higher compared with the colon (Figure 3*B*), we observed stepwise increase in Gdnf mRNA levels from WT to *Gdnf*^*WT/Hyper*^ and *Gdnf*^*Hyper/Hyper*^ mice as expected for derepression with Gdnf 3’UTR replacement allele. In the colon on the contrary we observed no difference in Gdnf mRNA levels between the genotypes, suggesting an inhibitory role for miR-9 and miR-133b on Gdnf mRNA levels. Expression levels for all studied microRNAs relative to snoRNA202 are presented in the Figure 3*D-F*.

### The Effect of GDNF on Enteric Ganglia Size and the Number of Nitrergic Neurons Is GDNF Dose-dependent

**Figure 4:**
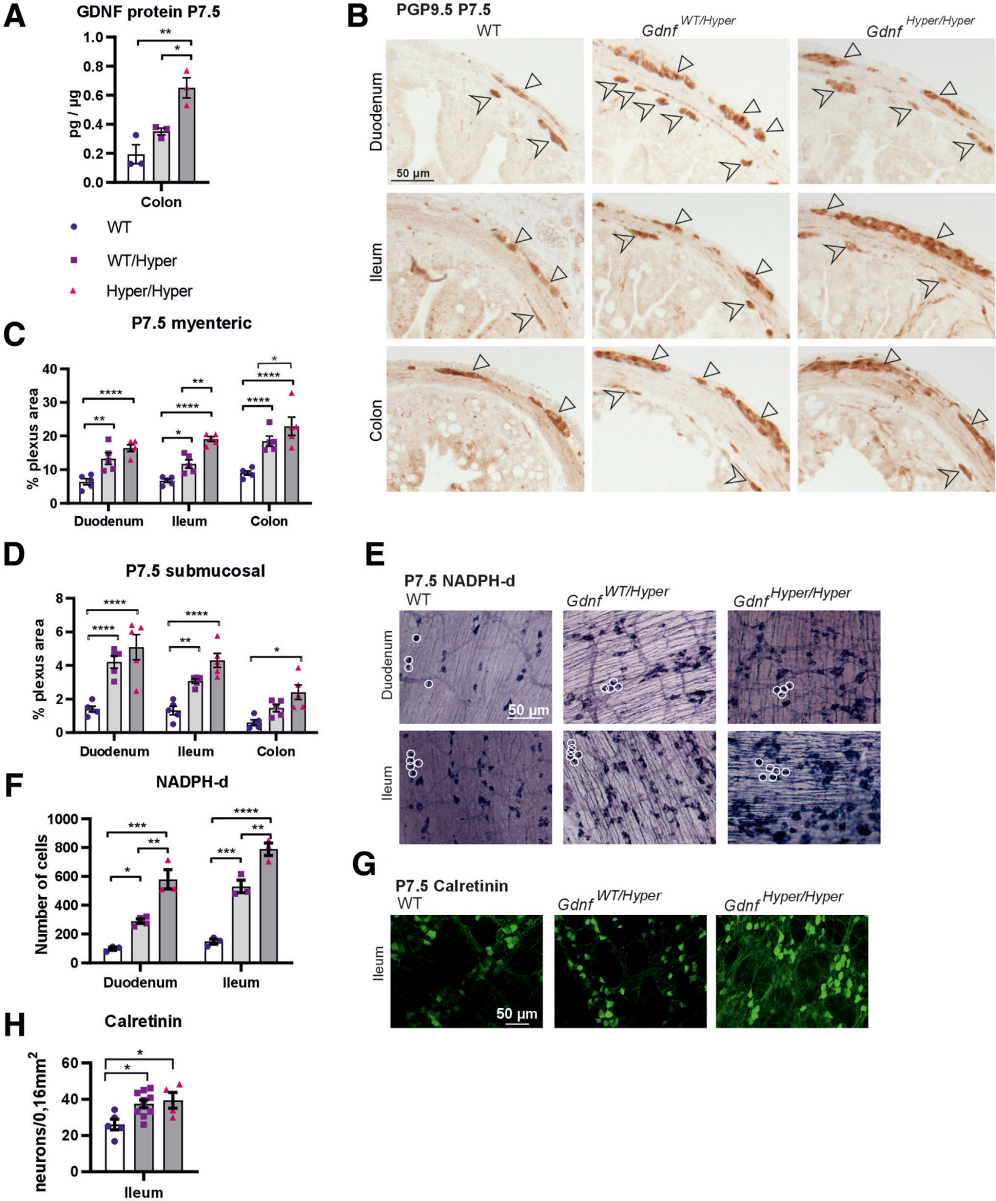
**Analysis of 3’UTR controlled GDNF dose effect on ENS size and composition in GDNF hypermorph mice.** (*A*) Analysis of GDNF protein level (n = 3; ∗*P* < .05; ∗∗*P* < .01) at P7.5 in the colon in indicated genotypes. (*B*) Representative images of pan-neuronal marker PGP9.5 staining from the duodenum, ileum, and colon. The neuronal area is increased in both *Gdnf*^*WT/Hyper*^ and *Gdnf*^*Hyper/Hyper*^ mice both in the (*C*) myenteric (n = 5; ∗*P* < .05; ∗∗*P* < .01; ∗∗∗∗*P* < .0001) and (*D*) submucosal (n = 5; ∗*P* < .05; ∗∗*P* < .01; ∗∗∗∗*P* < .0001) plexus. Myenteric neuronal area is increased between *Gdnf*^*Hyper/Hyper*^ versus *Gdnf*^*WT/Hyper*^ in the ileum (∗∗*P* < .01) and in the colon (∗*P* < .05). (*E*) Representative images of whole mount LMMP preparations of the duodenum and ileum stained with NADPH diaphorase histochemistry for nitrergic neurons. (*F*) The number of neurons is increased both in *Gdnf*^*WT/Hyper*^ and *Gdnf*^*Hyper/Hyper*^ mice compared with WT littermates and also in *Gdnf*^*Hyper/Hyper*^ versus *Gdnf*^*WT/Hyper*^ mice in the duodenum (n = 3–4; ∗*P* < .05; ∗∗*P* < .01; ∗∗∗*P* < .001) and ileum (n = 3; ∗∗*P* < .01; ∗∗∗*P* < .001; ∗∗∗∗*P* < .0001). (*G*) Representative images of whole mount LMMP preparations of the ileum stained with anti-calretinin for calretinin containing neurons. (*H*) The number of calretinin neurons is increased both in *Gdnf*^*WT/Hyper*^ and *Gdnf*^*Hyper/Hyper*^ mice compared with WT littermates (n = 4–10; ∗*P* < .05).

To study how endogenous GDNF levels affect the postnatal gut and its nervous system, we chose P7.5 as a timepoint for analysis. Homozygous *Gdnf*^*Hyper/Hyper*^ mice die usually by the age of P10–14 because of miniature kidneys and a malformed urogenital tract induced by abnormal migration and development of GDNF receptor bearing cells in urogenital block^14^^,^^16^ making analysis of homozygosity in adult mice impossible. However, most of the enteric neuronal subtypes are already born by P7.5.^17^^,^^18^ Similar to the adult colon (Figure 1*G*), GDNF protein was upregulated in *Gdnf*^*Hyper/Hyper*^ and *Gdnf*^*WT/Hype*r^ mice compared with WT littermates in an allele dose-dependent manner (Figure 4*A*).

Analysis of the ENS in *Gdnf*^*WT/Hyper*^ and *Gdnf*^*Hyper/Hyper*^ mice revealed that the area covered by pan-neuronal staining was increased in the duodenum, ileum, and colon. In the myenteric plexus, the ganglia size was increased by 2- to 3-fold (Figure 4*B*, *C*) and in the submucosal plexus, 3- to 3.5-fold (Figure 4*D*) at P7.5. Compared with the ganglia of *Gdnf*^*WT/Hyper*^ mice, in *Gdnf*^*Hyper/Hyper*^ mice, ganglia appeared larger, suggesting that GDNF affects ganglia size in a dose-dependent manner.

Next, we analyzed how the major functional myenteric neuronal populations are affected in *Gdnf*^*Hyper*^ mice. Analysis of nitrergic neurons using NADPH diaphorase revealed a GDNF dose-dependent effect on the number of nitrergic neurons: a 5- to 6-fold and 3- to 3.5-fold increase in NADPH^+^ cell numbers in *Gdnf*^*Hyper/Hyper*^ and *Gdnf*^*WT/Hyper*^ mice, respectively (Figure 4*E*, *F*). Quantification of myenteric neurons with an antibody against calretinin revealed an approximate 40% increase in calretinin-positive neurons (Figure 4*G*, *H*) with no difference between homozygotes and heterozygotes. This indicates that GDNF, when increased via abolishing negative regulation through altering its 3’UTR, has only a moderate effect on calretinin-positive neurons, with a maximum effect reached already in the heterozygous *Gdnf*^*WT/Hyper*^ mice.

**Figure 5:**
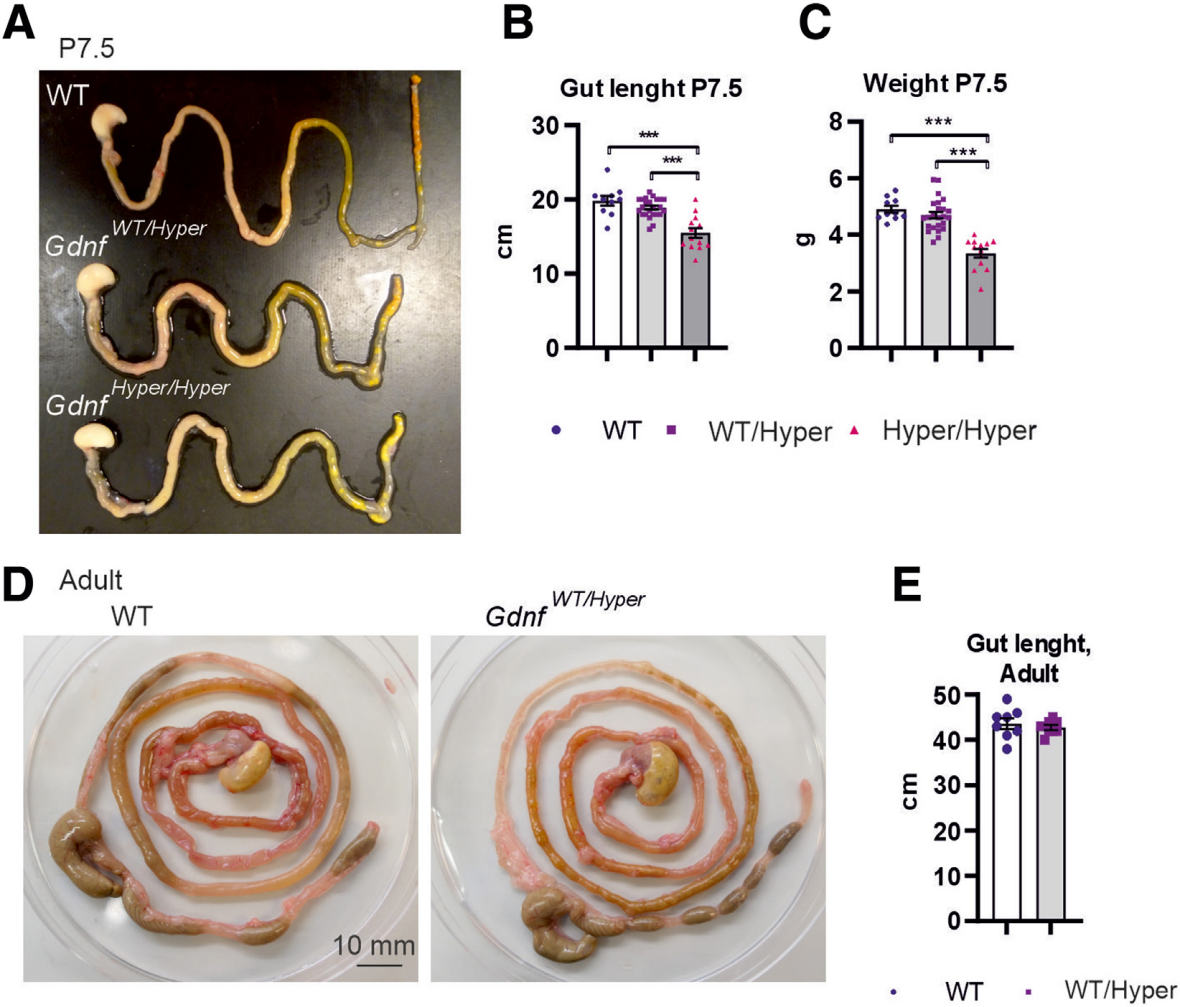
**Analysis of gut morphology in GDNF hypermorph mice.** (*A*) Representative image of P7.5 *Gdnf*^*Hyper/Hyper*^ and *Gdnf*^*WT/Hyper*^ mouse gut compared with WT littermate. (*B*) The total gut length is shorter in *Gdnf*^*Hyper/Hyper*^ mice (n = 10–24; ∗∗∗*P* < .001). (*C*) P7.5 *Gdnf*^*Hyper/Hyper*^ mice are smaller than their littermates because of kidney problems^14^ (n = 10–24; ∗∗∗*P* < .001). (*D*) Representative images of GI tracts from adult *Gdnf*^*WT/Hyper*^ mice. (*E*) Analysis of gut length in adult mice (n = 8).

Although we observed robust changes in ENS structure, there were no differences in the gross morphology of *Gdnf*^*Hyper*^ mouse guts at P7.5 compared with WT (Figure 5*A*). Total gut length in *Gdnf*^*Hyper/Hyper*^ mice was found to be shorter (Figure 5*B*), but this is in proportion to their reduced bodyweight (Figure 5*C*) stemming from overall failure to thrive at P7.5 because of kidney failure.^14^^,^^16^ Similarly, in adult *Gdnf*^*WT/Hyper*^ mice, no difference in total gut length was observed compared with WT littermate gender-matched control animals (Figure 5*D*, *E*).

### Conditional *Gdnf* 3’UTR Replacement Results in Similar Alterations in the Enteric Nervous System as in *Gdnf*^*WT/Hyper*^ Mice

In the *Gdnf*^*Hyper*^ allele, a marker gene encoding for drug resistance to puromycin and the thymidine kinase fusion protein is knocked-in to the *Gdnf* locus and replaces the native *Gdnf* 3’UTR (Figure 1*D1*).^14^ This “puroΔtk cassette” includes its own promoter and encodes for protein, whereas the transcription stop signal in this cassette is provided by the bovine growth hormone polyadenylation sequence (bGHpA). We cannot exclude that the puroΔtk cassette as a 3’UTR involves a gain of function phenotype beyond disrupting microRNA-mediated suppression of GDNF expression.

**Figure 6:**
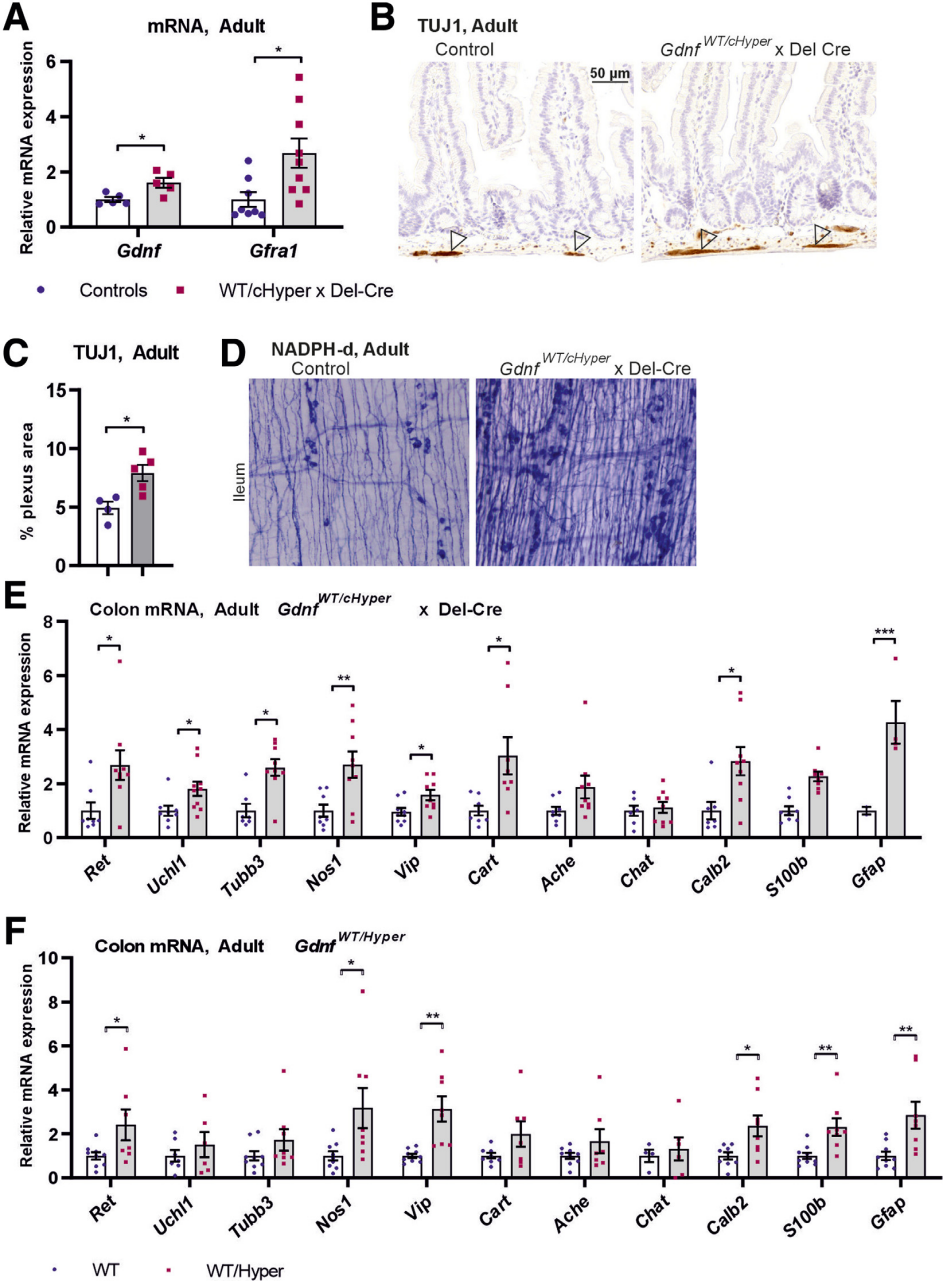
**Analysis of ENS in GDNF conditional hypermorph mice.** (*A*) qPCR analysis of mRNA levels of Gdnf (n = 5; ∗*P* < .05) and its receptor Gfra1 (n = 8–9; ∗*P* < .05) in adult *Gdnf*^*WT/cHyper*^ x Del-Cre mice. (*B*) Representative images of the pan-neuronal marker TUJ1 immunohistochemistry in adult conditional hypermorphic mice and control (–Cre [Cre negative] x *Gdnf*^*cHyper*^ and +Cre [Cre positive] x WT) littermates. (*C*) Quantification of myenteric plexus area using pan-neuronal marker TUJ1 immunohistochemistry staining (n = 4–5; ∗*P* < .05). (*D*) Representative images of NADPH diaphorase histochemistry in *Gdnf*^*WT/cHyper*^ x Del-Cre and control mice. *Gdnf*^*WT/cHyper*^ x Del-Cre mice have similar increase in NADPH diaphorase positive neurons as *Gdnf*^*WT/Hyper*^ mice (Figure 4*E*). (*E*) qPCR analysis of neuronal and glial markers in the colon of *Gdnf*^*WT/cHyper*^ x Del-Cre mice and control animals (n = 2–10; ∗*P* < .05; ∗∗*P* < .01; ∗∗∗*P* < .001). (*F*) Comparative analysis of the same genes as on *E* in the colon of *Gdnf*^*WT/Hyper*^ mice (n = 4–9; ∗*P* < .05; ∗∗*P* < .01.

Therefore, we next characterized the ENS in mice where the *Gdnf* 3’UTR is conditionally replaced with a bGHpA by germ-line active Cre (Del-Cre) (Figure 1*D2*).^15^ Quantitative polymerase chain reaction (qPCR) analysis of colon samples of adult *Gdnf*^*WT/cHyper*^ x Del-Cre mice (Figure 6*A*) displayed a similar increase in Gdnf and Gfra1 mRNA as in *Gdnf*^*WT/Hyper*^ mice (Figure 1*F*, *H*)

In *Gdnf*^*WT/cHyper*^ x Del-Cre mice we found an approximate 2-fold increase in the plexus area in the duodenum myenteric plexus (Figure 6*B*, *C*) similar to the duodenum myenteric plexus increase observed in *Gdnf*^*WT/Hyper*^ mice (Figure 2*C*, *D*). NADPH diaphorase histologic analysis on *Gdnf*^*WT/cHyper*^ x Del-Cre mice revealed increase in NADPH diaphorase-positive neurons comparable with what was observed in *Gdnf*^*WT/Hyper*^ mice (Figures 6*D* and 4*E*). Analysis of mRNA levels of different neuronal markers with qPCR revealed very similar changes in both *Gdnf*^*WT/cHyper*^ x Del-Cre (Figure 6*E*) and *Gdnf*^*WT/Hyper*^ (Figure 6*F*) mice.

### GDNF Levels Alter Gastrointestinal Function in Young and Old Mice

Next, we analyzed if and how a 2- to 4-fold increase in endogenous GDNF and the associated enhanced nitrergic compartment affects gut function.

**Figure 7:**
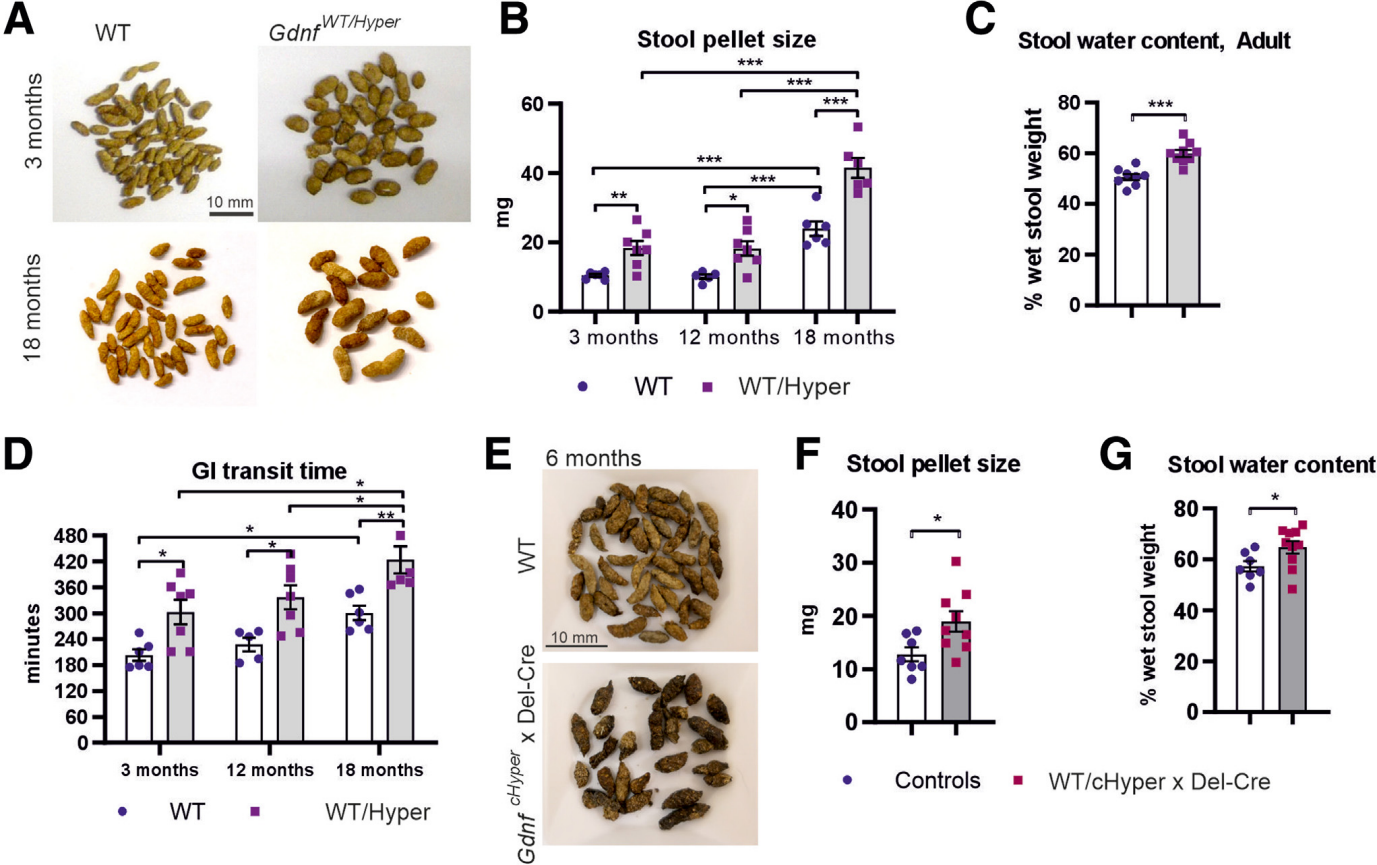
**Analysis of GI tract function in mice with different GDNF levels.** (*A*) Representative images of the stool pellets. (*B*) The average weight of a stool pellet was calculated from a 24 hours stool collection. The dry stool size is bigger in *Gdnf*^*WT/Hyper*^ mice at 3 months (∗∗*P* < .01; n = 6–7), 12 months (∗*P* < .05; n = 5–7), and 18 months (∗∗∗*P* < .001; n = 6). (*C*) The water percentage was calculated for fresh samples after 10 minutes of collection. The water content in adult *Gdnf*^*WT/Hyper*^ mouse stool samples is increased (n = 8–9; ∗∗∗*P* < .001). (*D*) A bolus of medicinal carbon was given to adult mice at different ages. The gastrointestinal transit time is slowed in the *Gdnf*^*WT/Hyper*^ mouse at 3 months (∗*P* < .05; n = 6–7), 12 months (∗*P* < .05; n = 5–7), and 18 months (∗∗*P* < .01; n = 6). (*E*) Representative images of stool pellets of GDNF conditional Hypermorph mice (*Gdnf*^*WT/cHyper*^ x Del-Cre) and control animals. (*F*) Stool size (n = 7–9; ∗*P* ≤ .05) and (*G*) stool water content (n = 7–10; ∗*P* < .05) are significantly increased in *Gdnf*^*WT/cHyper*^ x Del-Cre mice compared with control animals.

Analysis of young, middle aged, and old WT and *Gdnf*^*WT/Hyper*^ mice revealed that stool pellet size in young *Gdnf*^*WT/Hyper*^ mice is increased (Figure 7*A*, *B*) and that in WT mice, stool pellet size increases by about 2-fold on normal aging (Figure 7*B*). In *Gdnf*^*WT/Hyper*^ mice, the increase in stool pellet size was proportional until 18 months of age when the study was concluded (Figure 7*B*). Measurements of stool water content revealed that the stool of *Gdnf*^*WT/Hyper*^ mice contains about 20% more water relative to control animals (Figure 7*C*).

Next, we measured gastrointestinal (GI) transit time at 3, 12, and 18 months of age. We found that relative to littermate control animals, the total intestinal transit time in *Gdnf*^*WT/Hyper*^ mice is increased by about 30% at all ages (Figure 7*D*). In line with a previous report,^19^ we found that normal aging induces about 30% increase in the GI transit time in WT mice (Figure 7*D*). In *Gdnf*^*WT/Hyper*^ mice, the age-related increase in GI transit time was proportional to the ∼30% increase observed in young mice (Figure 7*D*).

Analysis of stool pellet size and stool water content of the *Gdnf*^*WT/cHyper*^ x Del-Cre mice revealed similar increases as found in *Gdnf*^*WT/Hyper*^ mice (Figure 7*E-G*), which aligns with the observed similar changes in ENS composition (Figure 4*B-F*, Figure 6).

### Gut Monoamine Levels Are Not Affected by GDNF Levels

**Figure 8:**
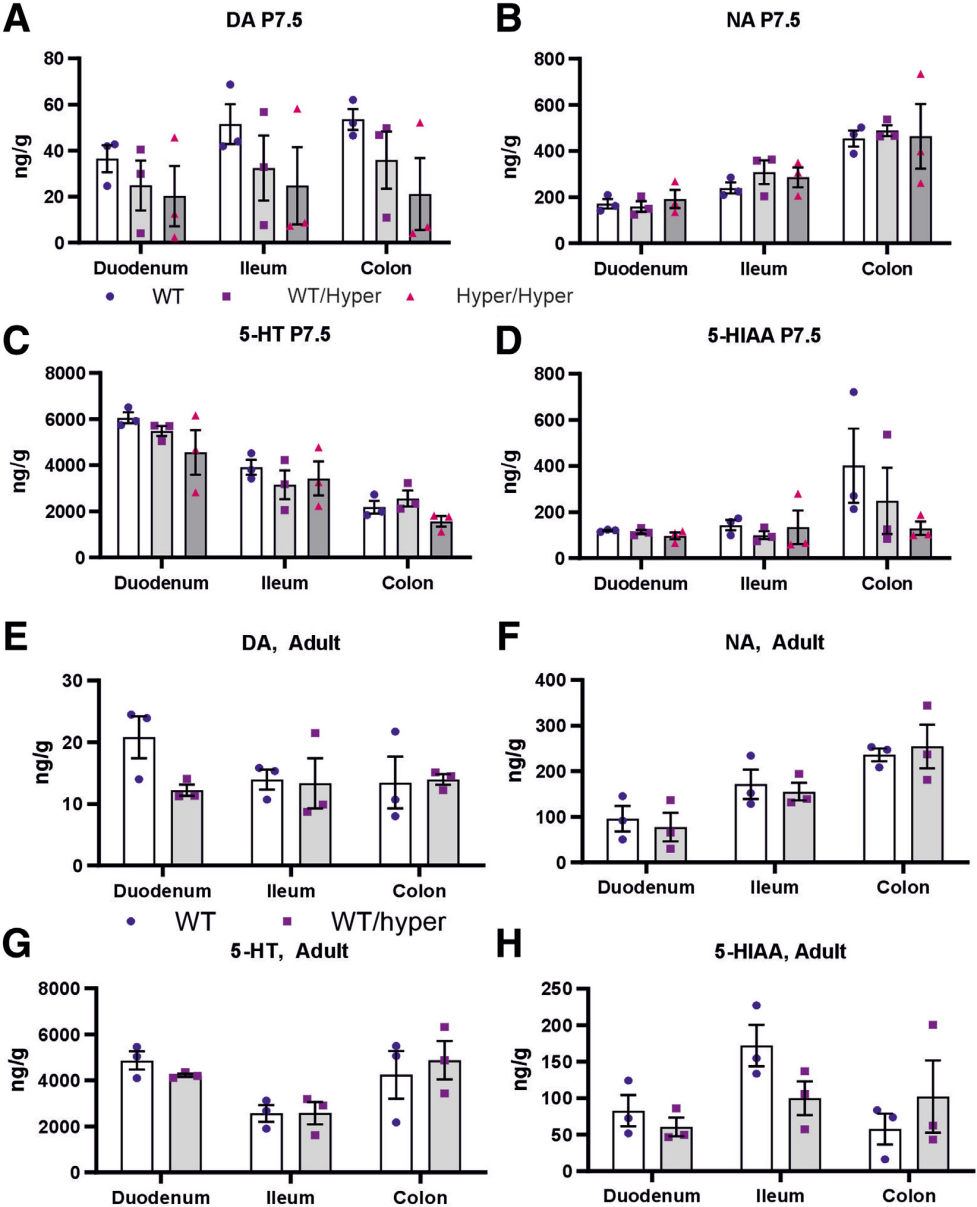
**Analysis of monoamines in GDNF hypermorph mice.** (*A-H*) Neurotransmitter levels of dopamine (DA), noradrenaline (NA), serotonin (5-HT), and its metabolite 5-hydroxyindoleacetic acid (5-HIAA) as measured by high-performance liquid chromatography in the indicated gut regions in P7.5 and in adult mice are not changed (n = 3).

In the brain, both ectopic and endogenous GDNF increases elicit strong dopamine level- and function-enhancing effects.^14^^,^^15^ Monoamines are known to modulate gut transit time, and the levels of serotonin are especially abundant in the gut. High-performance liquid chromatography analysis of dopamine, noradrenaline, serotonin, and serotonin metabolite 5-HIAA revealed no differences in the intestine between genotypes at P7.5 and in adult mice (Figure 8*A-H*), suggesting that GDNF levels, controlled via 3’UTR editing, do not affect gut monoamine levels.

### Increase in Endogenous GDNF Levels Is Associated with Mild Epithelial Barrier Function Disruption and Increased Inflammatory Tone in Mice

**Figure 9:**
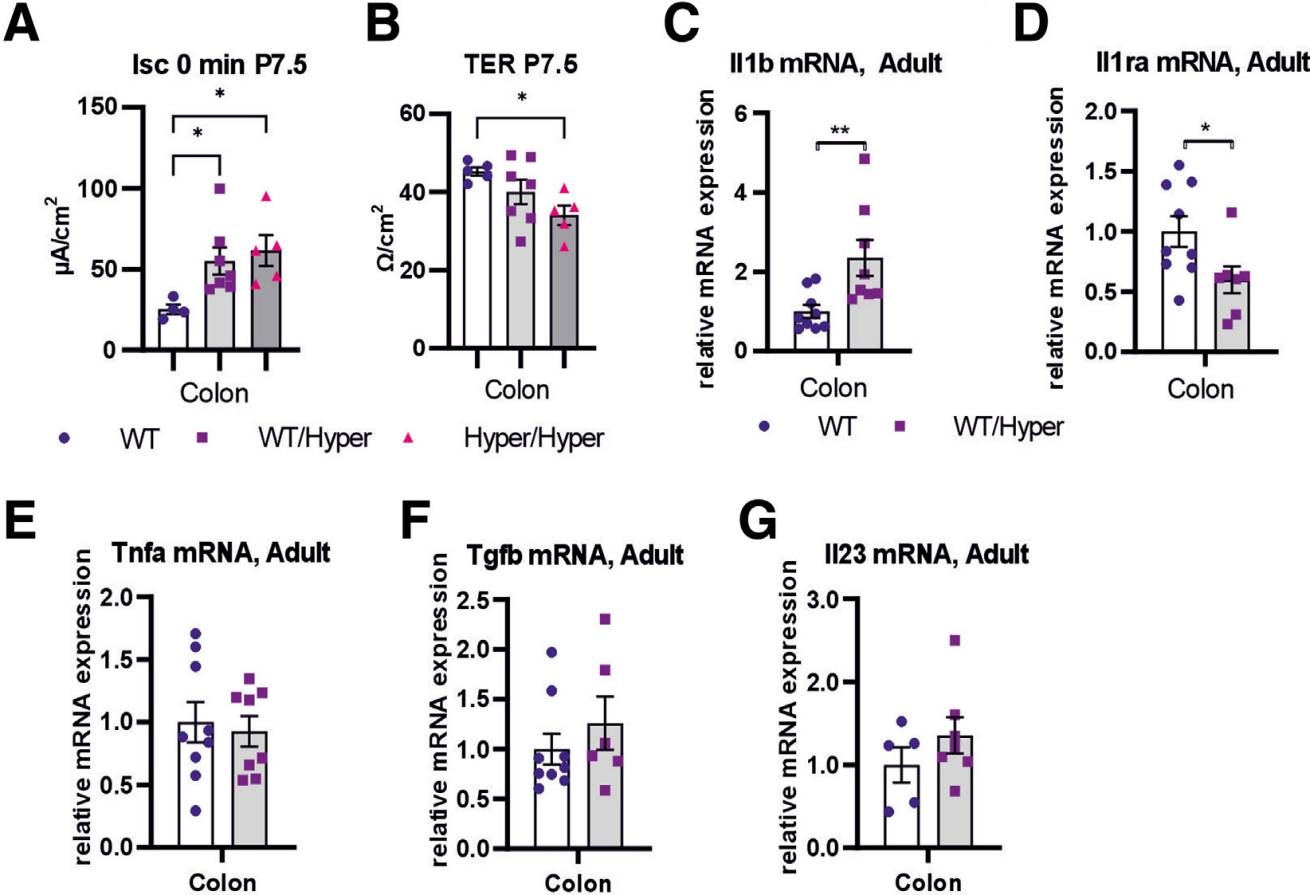
**Analysis of epithelial function and cytokines in GDNF hypermorph mice.** (*A*) At P7.5 the colon of *Gdnf*^*WT/Hyper*^ and *Gdnf*^*Hyper/Hyper*^ mice display increase in short circuit current (Isc) compared with WT (n = 4–7; ∗*P* < .05). (*B*) TER is significantly decreased in *Gdnf*^*Hyper/Hyper*^ mice (P7.5) compared with WT (n = 5–7; ∗*P* < .05). (*C*, *D*) In the adult *Gdnf*^*WT/Hyper*^ mice colon, mRNA levels of proinflammatory (*C*) IL1b is upregulated and IL1 inhibitor Il1ra (*D*) mRNA is downregulated compared with WT littermates (n = 7–9; ∗*P* < .05; ∗∗*P* < .01). (*E-G*) Cytokine Tnf, Tgfb, and Il23 mRNA levels in the colon are comparable between the genotypes (n = 5–9).

Enteric neurons that have cell bodies in the submucosa innervate the epithelium and regulate epithelial function.^20^ GDNF hypermorphic mice have more enteric submucosal neurons (Figure 4*D*) and an increase in stool water content (Figure 7*C*), which may relate to altered epithelial function. Analysis of the short circuit current (Isc) revealed a significant increase in GDNF hypermorphic mice compared with WT mice (Figure 9*A*), indicating increased net ion transport across the epithelium. In addition, a significant decrease in transepithelial electrical resistance (TER) was observed in *Gdnf*^*Hyper/Hyper*^ mice (Figure 9*B*).

Cytokine levels determine gut inflammatory tone and can modulate various GI tract functions and pathologic processes.^21^ Analysis of mRNA levels of Tnfa, Tgfb, Il23, Il1b, and its inhibitor Il1ra in GDNF hypermorphic mice revealed approximately a 2.5-fold increase in Il1b mRNA levels, whereas the mRNA levels of Il1ra, a secreted natural inhibitor of IL1B, were decreased (Figure 9*C*, *D*). The expression levels of Tnfa, Tgfb, and Il23 were not significantly altered (Figure 9*E-G*).

### GDNF Regulates *Nos1* Expression in Embryonic Enteric Neurons Via Transcription Factor ETV1

**Figure 10:**
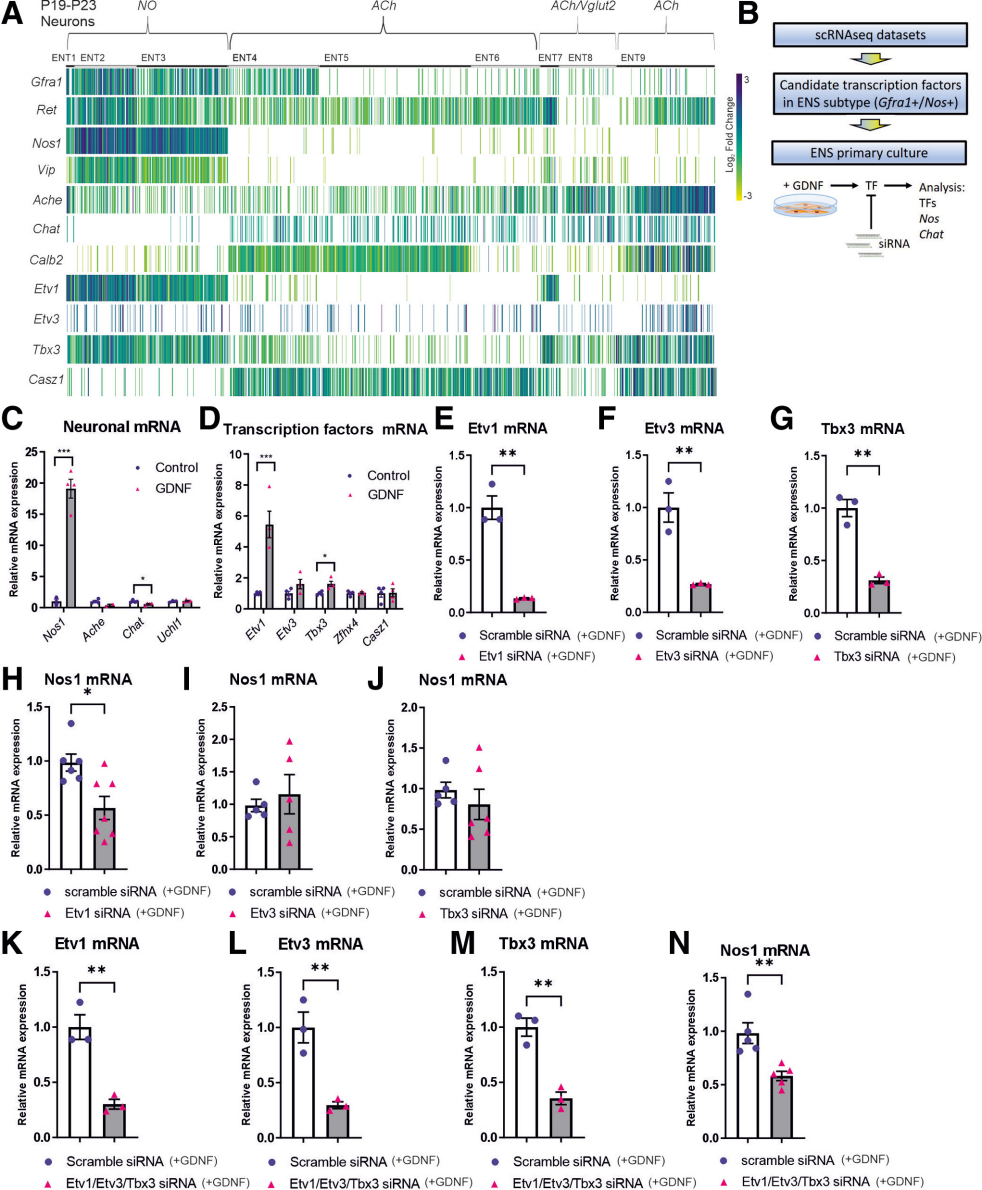
**Analysis of GDNF, its receptors, and downstream effectors expression and function.** (*A*) Data gathered from open single-cell RNA sequencing database,^22^ ENT1-ENT9 represent different clusters of neurons. NO, nitric oxide; ACh, acetylcholine; Vglut2, vesicular glutamate transporter 2. (*B*) Workflow for analysis of GDNF downstream effectors, E12.5 mouse guts were used for primary cultures. (*C*) Addition of recombinant GDNF protein upregulates Nos1 mRNA, but not Ache, Chat, or Uchl1 mRNA expression in enteric neuron progenitor cells (n = 3–4; ∗∗∗*P* < .001). (*D*) Addition of GDNF upregulates Etv1 and Tbx3 mRNA levels (n = 3–4; ∗*P* < .05; ∗∗∗*P* < .001). (*E-G*) Transfection with siRNAs targeting Etv1, Etv3, and Tbx3 causes a significant downregulation in corresponding mRNA expression in enteric neuron progenitor cells (n = 3; ∗∗*P* < .01). (*H-J*) knockdown of (*H*) Etv1 results in downregulation of Nos1 mRNA expression in enteric neuron progenitor cells, whereas knockdown of Etv3 and Tbx3 does not (*I*, *J*) (n = 5–7; ∗*P* < .05). (*K-M*) Transfection with combination of siRNAs targeting Etv1, Etv3, and Tbx3 results in downregulation of (*K*) Etv1, (*L*) Etv3, and (*M*) Tbx3 mRNA levels (n = 3; ∗∗*P* < .01). (N) Knockdown of Etv1, Etv3, and Tbx3 mRNA levels results in significant downregulation of Nos1 expression (n = 5; ∗∗*P* < .01), similar to what is observed when Etv1 alone is downregulated (*H*).

Next, we explored how GDNF induces nitrergic differentiation. Enteric nitrergic neurons coexpress GDNF receptors Gfra1 and Ret (http://loom.linnarssonlab.org/) (Figure 10*A*).^22^ We took advantage of 2 published single-cell RNAseq analyses^22^^,^^23^ and identified Etv1 and Tbx3 (http://loom.linnarssonlab.org/)^22^ and Zhfx4 and Tbx3 (from Wright et al^23^) as candidate transcription factors (TFs) that were enriched in nitrergic neurons. Recently, Etv1 and Tbx3 were identified as regulatory candidate genes of neurogenic branching during ENS development.^24^ TFs Casz1, which is expressed in cholinergic enteric neurons, and Etv3, which shows minimal expression in enteric neurons were included as putative negative control animals (Figure 10*A*). The workflow for the experiment is shown in Figure 10*B*. Primary culture containing neuronal progenitors and enteric neurons were obtained from E12.5 mouse embryos and cultured in the presence of recombinant GDNF or vehicle for 24 hours. Recombinant GDNF induced up to 20-fold increase in Nos1 mRNA expression (Figure 10*C*). No significant upregulation in cholinergic markers Ache or Chat or in pan-neuronal marker Uchl1 was observed (Figure 10*C*). We then analyzed the expression of TFs after the addition of GDNF and observed significant upregulation in Etv1 and Tbx3 but not in Etv3 and Zhfx4 mRNA-levels (Figure 10*D*). Casz1, a TF that denotes cholinergic lineage,^22^, ^23^, ^24^ was not significantly upregulated.

Next, we examined how GDNF-induced TFs affect GDNF-mediated nitrergic differentiation. We transfected our in vitro cultures with siRNAs targeting Etv1, Etv3, and Tbx3 and observed a 70%–85% reduction in TF expression (Figure 10*E-G*). We then transfected enteric neuronal progenitors with the aforementioned siRNAs or with scrambled siRNA control animals for 24 hours, after which point GDNF was added. We observed that knockdown of Etv1 (Figure 10*H*), but not Etv3 or Tbx3 (Figure 10*I, J*), caused approximately a 50% reduction in GDNF-induced *Nos1* expression. We also transfected enteric neuronal progenitors with siRNAs specific to Etv1, Etv3, and Tbx3 together, which resulted in about 70% reduction in Etv1, Etv3, and Tbx3 expression (Figure 10*K-M*). Compared with the knockdown of Etv1 alone (Figure 10*H*), knockdown of Etv1, Etv3, and Tbx3 together (Figure 10*N*) also resulted in similar reduction in GDNF-induced Nos1 expression, indicating that GDNF regulates nitrergic differentiation mainly by inducing Etv1 expression and via ETV1 function.

**Figure 11:**
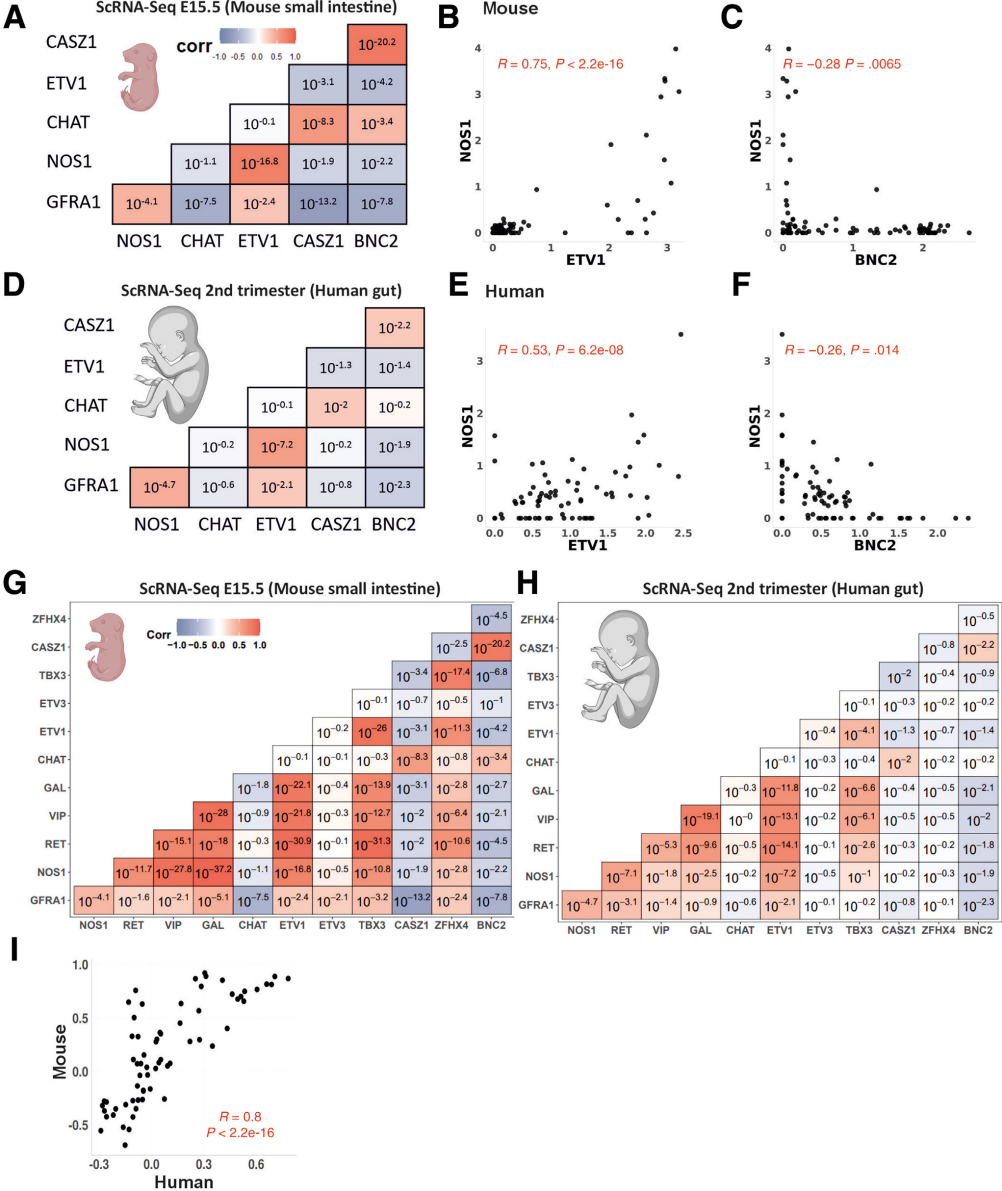
**GFRa1 mRNA levels positively correlate with ETV1 and nitrergic, but not cholinergic marker expression in developing mouse and human ENS.** (*A*) Pairwise correlation matrix for E15.5 mouse small intestine samples. (*B*, *C*) The Pearson correlation was used to determine linear relationships between levels of NOS1 and ETV1 and NOS1 and BNC2 in mouse small intestine (E15.5). (*D*) Pairwise correlation matrix for human second trimester gut samples. (*E*, *F*) Linear relationships between levels of NOS1 and ETV1 and NOS1 and BNC2 in human second trimester gut samples. (*G-I*) The pairwise correlation coefficients calculated for the set of GFRa1, NOS1, RET, VIP, GAL, CHAT, ETV1, ETV3, TBX3, CASZ1, ZHFX4, and BNC2 genes in both (*G*) mouse small intestine (E15.5) and (*H*) human second trimester gut samples revealed a consistent pattern of correlation, suggesting a high degree of similarity in the gene relationships between human and mouse (*I*) (*R =* 0.8; *P =* 2.2^-16^). The corr bar plot shows the range of correlations, with *dark blue* indicating the largest negative correlation and *dark red* indicating the highest positive correlation. The values in each cell are the *P* value. The scCorr package was used to compute correlation coefficients. Note the similarity in pattern between mouse and human samples (image created using biorender.com).

Next, we analyzed how GDNF signaling correlates with the levels of ETV1 and NOS1 in single neurons from midgestation onward using human and mouse single-cell sequencing data from the developing gut. Our results from the adult human colon, knock-in mouse models, and in vitro analysis predict that elevated GDNF signaling enhances ETV1 expression and that high ETV1 levels correlate with NOS1 expression in the same neurons. Analysis of scRNAseq datasets from E15.5 mouse (GSE149524)^24^ (Figure 11*A-C*) and postcoitum week 12–17 (second trimester) human small intestine (https://www.gutcellatlas.org)^25^ (Figure 11*D-F*) revealed strong positive correlations between GFRa1 and marker genes ETV1 and NOS1, but not CASZ1, BNC2, or CHAT levels in the same neurons in both mouse and human datasets, as our results predicted. Similarly, the correlation pattern (GFRa1, NOS1, RET, VIP, GAL, CHAT, ETV1, ETV3, TBX3, CASZ1, ZHFX4, BNC2) between mouse and human datasets aligned closely (*R =* 0.8; *P =* 2.2^-16^) (Figure 11*G-I*).

## Discussion

The 3’UTR controls gene expression at the posttranscriptional level.^26^ In line with this, we previously found that Gdnf mRNA expression in *Gdnf*^*Hyper*^ mice is increased but not spatially altered in the brain, spinal cord, kidney, and testis, allowing the study of the effect of increased endogenous GDNF expression.^14^, ^15^, ^16^ Interestingly, and perhaps as expected for a pleiotropic morphogen, such as GDNF, results from gene deletion most often do not predict the effects of increased expression likely because of various reasons. For example, deletion of GDNF in the brain enhances dopamine transporter function^27^ as does a 2- to 3-fold increase in endogenous GDNF expression.^14^ During kidney development, GDNF deletion leads to tiny or even complete agenesis of the kidneys,^28^ but an increase in endogenous GDNF expression does not result in larger kidneys as might be expected. Instead, an increase in endogenous GDNF expression results in hypotrophic and malformed kidneys^14^ because of local effects of elevated GDNF levels on ureteric bud tip cells.^16^ In the gut, complete GDNF deletion leads to an absence of the ENS distal to the stomach,^28^ whereas *Gdnf*^*+/-*^ mice have a 50%–60% reduction in the myenteric neurons without a selective loss in number of nitrergic neurons.^29^

How endogenous GDNF levels normally vary, what mechanisms regulate GDNF levels, and if and how this affects ENS formation and function is currently not well understood, inspiring the current study. Ectopic GDNF expression in the gut may be helpful,^12^ but does not address these questions. It provides information on ectopic GDNF function at the specific time, amount, and location determined by the expression system.

It is well known that GI tract function varies remarkably between the individuals.^30^ The ENS is the key regulator of diverse GI tract functions including gut transit time, fluid and electrolyte secretion, and inflammatory tone.^7^ Only recently it has emerged that the ENS ganglia size varies at least 2- to 3-fold between individuals,^1^, ^2^, ^3^, ^4^, ^5^, ^6^ but mechanisms driving this variation have remained obscure.

**Figure 12:**
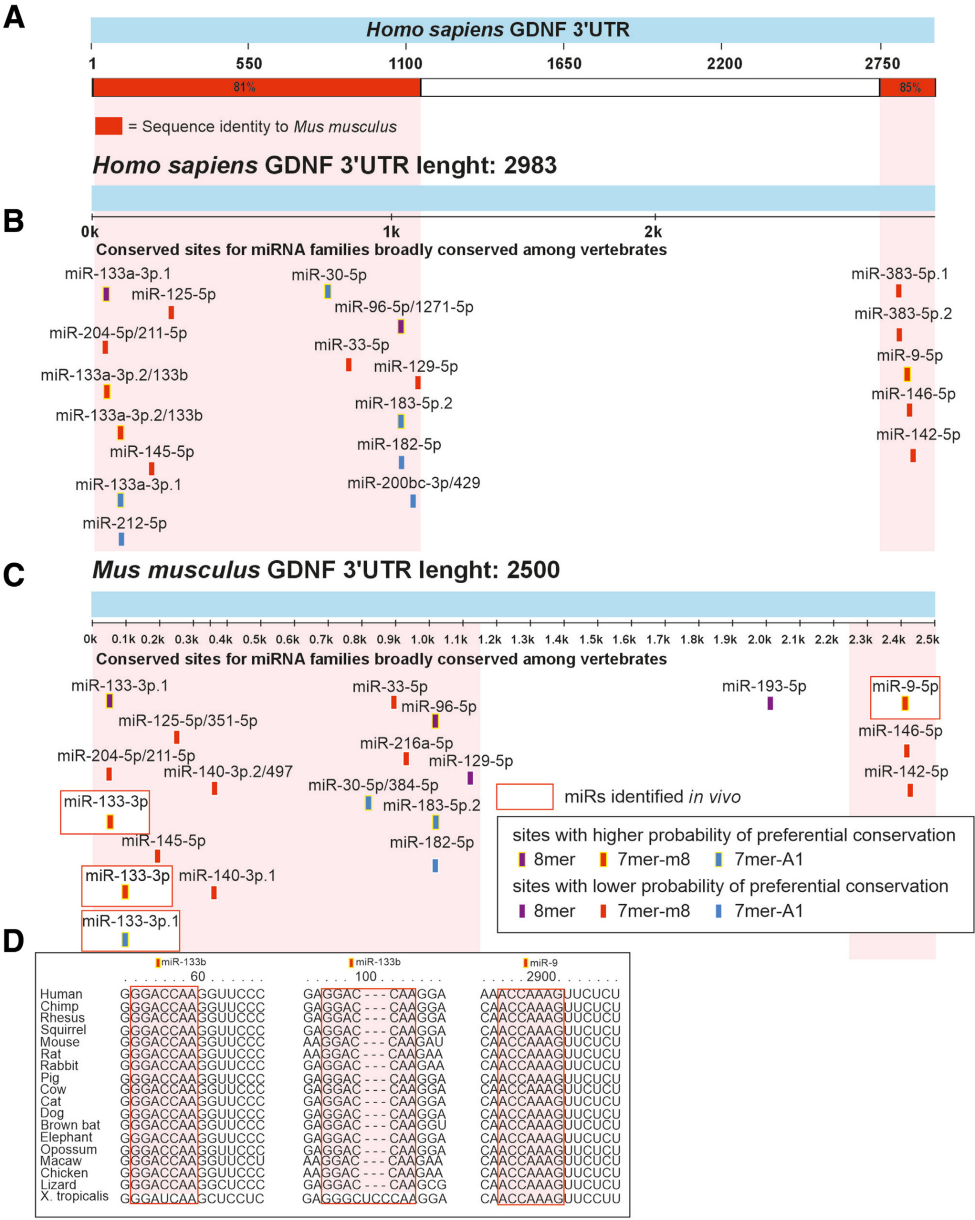
**GDNF 3’UTR and binding sites for miR-133b and miR-9 are evolutionarily conserved in vertebrate evolution.** (*A*) Schematic diagram showing >80% sequence identity between human (Homo sapiens) and mouse (Mus musculus) GDNF 3’UTR regions (*red*). (*B*) miRNA binding sites in human and (*C*) mouse GDNF 3’UTR that are broadly conserved among vertebrates. miR-9 and miR-133b that directly bind GDNF 3’UTR and via this interaction negatively regulate GDNF levels^14^ are highlighted with *red rectangles*. (*D*) Seed sequences (*colored boxes*) in GDNF 3’UTR for miR-133b and miR-9 are conserved among vertebrates, estimated evolutionary distance between humans and *Xenopus tropicalis* is >360 million years.^32^

Here we analyzed how endogenous GDNF levels vary between individuals and if and how this influences ENS composition, size, and function. We found that in the adult human colon, GDNF mRNA levels vary at least up to 5-fold among individuals and that GDNF levels correlate with pan-neuronal and nitrergic, but not cholinergic, marker expression (http://gepia2.cancer-pku.cn/). In mice, a similar upregulation of endogenous GDNF expression at the posttranscriptional level via Gdnf 3’UTR replacement warranting increased expression only in naturally GDNF-expressing cells resulted in a similar increase in nitrergic marker gene expression and up to a 3-fold increase in ENS ganglia size in a GDNF dose-dependent manner. Assessment of microRNAs miR-133b and miR-9, which bind to GDNF-encoding mRNA^31^ and negatively regulate GDNF expression via defined binding sites,^14^ revealed a negative correlation between GDNF levels and their expression in the developing intestine. This suggests, although does not conclusively demonstrate, their role in negatively regulating GDNF levels during ENS development. GDNF 3ʹUTR is highly conserved in evolution (Figure 12*A*) and miR-133 and miR-9 binding sites in GDNF 3ʹUTR are conserved from African clawed frog *Xenopus tropicalis* onward from about 360 million years ago,^32^ pointing to important biologic function (Figure 12*B-D*). Analysis of different knock-in alleles in mice with GDNF 3’UTR replacement showed very similar results to our findings in human samples, suggesting that the observed effects on the ENS size, nitrergic composition, and function indeed may derive from normal variation in GDNF levels.

Our analysis of TFs in cells positive for GDNF receptors GFRa1 and RET^22^, ^23^, ^24^ revealed that GDNF both induces the expression of and at least, in part, requires the function of ETV1 to enhance nitrergic marker expression in the developing ENS in vitro.

In the developing gut, GDNF is not expressed in the same cells as GFRa1/RET. Thus, quantitative correlation analysis using the human and mouse developing gut single-cell RNAseq data is not possible. However, we^15^ and others^33^^,^^34^ have shown that GDNF signaling upregulates GFRa1 expression in various non-neuronal and neuronal tissues. Higher GFRa1 levels, in turn, likely allow stronger GDNF signaling. We found that GDNF levels positively correlate with GFRa1 and NOS1 levels in the adult human colon (http://gepia2.cancer-pku.cn/) and that in the single-cell analysis GFRa1 levels positively correlate with ETV1 and nitrergic, but not cholinergic, marker expression in developing mouse (GSE149524)^24^ and human ENS (https://www.gutcellatlas.org)^25^ in the same neurons. Collectively, our data suggest that GDNF levels, regulated at least in mice by its 3’UTR, predominantly affect the nitrergic compartment size in the ENS at least in part via inducing ETV1 expression and via ETV1 function. The latter is revealing, because ETV1 has been identified as a marker of nitrergic lineage in both the mouse and human developing ENS in multiple scRNAseq studies.^23^, ^24^, ^25^^,^^35^ Moreover, it was recently reported that *Ret* deficiency during development leads to loss of nitrergic neuronal population without affecting cholinergic compartment,^36^ which aligns with our results. Our work reveals the functional importance of ETV1 as a regulator of nitrergic neuron formation and places GDNF signaling upstream of ETV1 function.

It is feasible to hypothesize that specific miR expression levels can vary between individuals during ENS development, providing one possible explanation for the observed variation in GDNF levels in the colon. Whether interindividual variation in GDNF levels in humans derives from differences in 3’UTR regulation or from other mechanisms awaits future studies.

**Figure 13:**
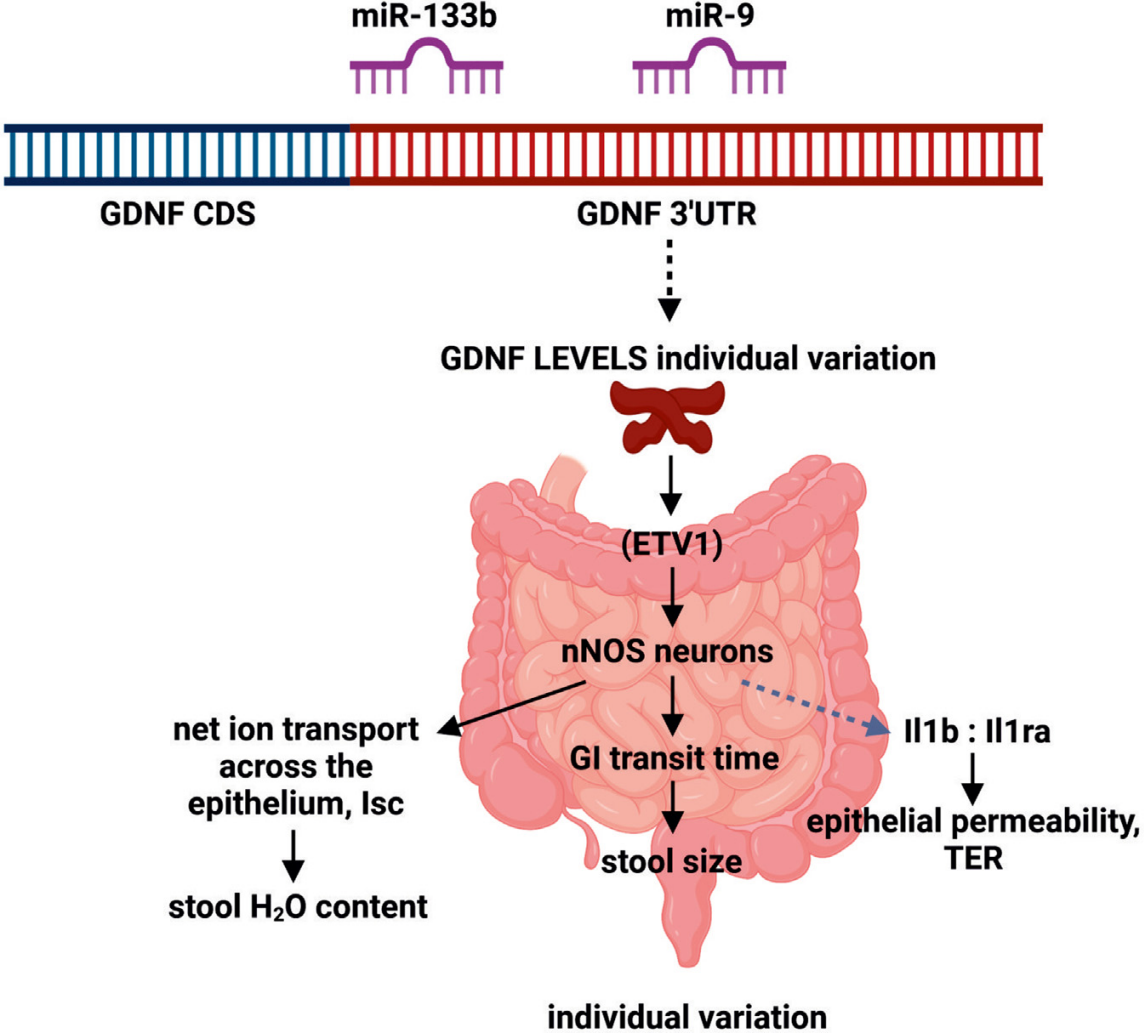
**Graphical summary of findings and proposed sequence of outcomes.** MiR-133b and miR-9 binding sites in GDNF 3’UTR are evolutionarily conserved for >360 million years (Figure 12), they bind directly to GDNF mRNA and regulate GDNF expression levels.^14^ The expression of miR-133b and miR-9 inversely correlate with GDNF expression in the developing gut (Figure 3*B*, *C*) as expected, providing one possible mechanism (*black dotted line*) for the observed variation in GDNF levels among humans (Figure 1*A*). GDNF levels regulate nitrergic (nNOS) neuron lineage at least in part via transcription factor ETV1. nNOS neurons are known to slow down the GI tract transit time^37^ resulting in larger stool size. Nitric oxide (NO) released from nNOS neurons also functions as secretomotor neurotransmitter^39^ and nNOS neurons have been shown to increase chloride secretion likely explaining observed increase in stool water content.^38^ NO also increases Il1b to Il1ra ratio by increasing the production of IL1B^41^, ^42^, ^43^ and by decreasing the production of IL1RA,^41^ indicated by *grey dotted line* to reflect less well-established causality. IL1B signaling increases epithelial permeability (leaky gut) which may increase the risk of inflammation in the gut^44^ (image created using biorender.com).

At the functional level, we observed that GDNF levels regulate gut transit time, stool size, and water content to a degree that is likely in the normal variation range observed in humans. It is well known that nitrergic neurons have inhibitory effect on the gut motility.^37^ Slower gut motility would in turn allow larger stool formation. In addition to regulating motility, nitric oxide–containing neurons have prosecretory function resulting in increased chloride secretion, which increases stool water content.^38^^,^^39^ We did not observe any obvious side effects or other effects throughout adulthood and on aging in mice with various GDNF levels until the study was concluded at 18 months of age. However, we did observe negative correlation between GDNF levels and epithelial integrity, which may be related to an increase in cytokine Il1b expression and reduced expression of its inhibitor Il1ra. Interestingly, a similar Il1b/Il1ra disbalance is believed to contribute to inflammatory bowel disease^40^ where inflammation may contribute to the reduction in epithelial integrity. The relationship between the ENS, epithelial integrity, and Il1b/Il1ra expression and possible predisposition to inflammatory bowel disease remains an interesting topic for future studies. A summary of our findings is presented in Figure 13 and in visual abstract.

Improved understanding of ENS development is critical for gaining new insights into common GI disorders, such as irritable bowel syndrome, inflammatory bowel disease, and chronic constipation,^7^ and to increasing number of diseases with ENS involvement, such as Parkinson disease, Alzheimer disease, and various GI tract cancers.^7^^,^^8^ Our work provides new knowledge base and novel experimental models to analyze ENS involvement in those disorders and beyond.

## Materials and Methods

### Human Gut RNA Sequencing Analysis

The expression levels of GDNF and NOS1 genes across 41 samples of healthy adult human colon were obtained using the GEPIA2 database (Gene Expression Profiling Interactive Analysis), which is accessible at http://gepia2.cancer-pku.cn/. In GEPIA, gene expression levels are presented in the TPM (transcript per million) values in log2 scale. TPM is calculated using the formula:

TPMi=qili∑jqjlj×106,

where *q*_*i*_ denotes reads mapped to the transcript *i*, and *l*_*i*_ is the transcript length. This online tool offers swift and customizable functionalities, which integrates RNA sequencing data from data derived from The Cancer Genome Atlas (TCGA) and The Common Fund's Genotype-Tissue Expression (GTEx) sources.^13^ GEPIA2 was used to access samples from TCGA derived from healthy colon tissue and to compute the Pearson correlation coefficient among ENS markers within the context of normal colon samples.

To study single cells from humans in the developmental gut, the Gut Cell Atlas for humans (accessible at https://www.gutcellatlas.org/), which is a full single cell RNA-seq dataset of 428,000 intestinal cells from fetal, pediatric, and adult donors, was used.^25^ We downloaded RAW.H5AD data and selected fetal neuronal cells (877 cells) from the second trimester (12–17 PCW) for our analysis. The Seurat R package^45^ was used to perform data normalization (LogNormalize). A key problem with single-cell RNA-seq data is the abundance of zero values, known as "dropout," which results in biased assessment of gene-gene correlations. To estimate the correlation between genes we used the R package scCorr version 01,^46^ which is a graph-based k-partitioning method, by merging transcriptomically similar cells. The pairwise Pearson correlation coefficients between GFRA1, NOS1, RET, VIP, GAL, CHAT, ETV1, ETV3, TBX3, CASZ1, ZFHX4, and BNC2 genes were calculated.

### Mouse Gut RNA Sequencing Analysis

Single-cell RNA-sequencing data of the embryonic mouse ENS of the small intestine was downloaded under the accession number GSE149524^24^ from the Gene Expression Omnibus (https://www.ncbi.nlm.nih.gov/geo/) database. They retrieved 3468 cells from E15.5 using Louvain clustering, of which 2476 are myenteric neurons cells from Branches A and B. The pairwise correlation using the scCorr package between the 11 aforementioned genes was calculated.

For single-cell RNA-sequencing analysis of the postnatal mouse ENS of the small intestine, the sequencing data used for this study are available at mousebrain.org,^22^ which contains a graphical interface.

### GDNF 3’UTR Sequence Identity Analysis

Blast (https://blast.ncbi.nlm.nih.gov/Blast.cgi) was used to evaluate the sequence identity of GDNF 3’UTR between human and mouse.

### Animals

*Gdnf* hypermorphic mice (*Gdnf*^*Hyper*^, Figure 1*D1*) were generated as previously described^14^ via replacing the native *Gdnf* 3’UTR with a puroΔtk cassette and bGHpA, which lack the microRNA binding sites of the WT *Gdnf* 3’UTR (colored bars on Figure 1*E*). Because *Gdnf*^*Hyper/Hyper*^ mice die before P14–P18 because of kidney malformations, heterozygous mice were used for analysis after P7.5. *Gdnf* conditional hypermorph (*Gdnf*^*cHyper*^) mice have been described previously.^15^ Briefly, in the cHyper allele, the bGHpA flanked by the FLEx system^47^ was placed in an inverted position immediately downstream of the stop codon of the *Gdnf* gene (Figure 1*D2*). After Cre recombination, the bGHpA is reversed, and the resulting mRNA lacks microRNA binding sites (Figure 1*D3*). In the Nestin-Cre mice^48^ used in this study, Cre recombinase is occasionally active in the germline, in which case it functions as a Deleter-Cre (Del-Cre) line.^49^ Here we used this property to generate hypermorphic GDNF mice (*Gdnf*^*cHyper*^ x Del-Cre). The mice were genotyped with routine PCR methods.^14^^,^^15^ *Gdnf*^*Hyper*^ and *Gdnf*^*cHyper*^ x Del-Cre mouse lines were maintained in 129Ola/ICR/C57bl6 mixed genetic background, housed in 12/12 light-dark cycle, and fed ad libitum. Experiments were performed during light cycle. For transgenic mouse lines, sibling littermate control animals were used for analysis. In experiments where *Gdnf*^*cHyper*^ x Del-Cre mice were used control group denotes litter matched –Cre (Cre negative) x *Gdnf*^*cHyper*^ mice and +Cre (Cre positive) x WT mice. A mouse was defined as “adult” when it was 2–6 months old, unless otherwise stated. When referring to the number of animals used in an experiment N number denotes number of animals per genotype. All animal experiments were authorized by the national Animal Experiment Board of Finland.

### Enzyme-Linked Immunosorbent Assay

#### Tissue Processing

Dissected tissues were immediately snap frozen. Samples from P7.5 and adult guts were homogenized with a bead homogenizer (Precellys) and E13.5 samples were homogenized manually.

#### Analysis of Protein Levels

Quantification of GDNF protein levels was performed using GDNF Emax Immunoassay (Promega) with acid treatment as suggested by the manufacturer. The 20–100 μg of total protein, measured with the DC Protein Assay (Bio-Rad), was loaded onto the enzyme-linked immunosorbent assay plate. All samples were analyzed in duplicates.

#### Tissue Processing for Histopathology and Immunohistochemistry

Paraffin sections or longitudinal muscle/myenteric plexus preparations (LMMP) were used for histologic preparations or immunohistochemistry. Comparable samples from the duodenum, ileum, and colon were subjected to analysis from all genotypes using littermate control animals. Samples ultimately processed into paraffin sections were fixed in 4% paraformaldehyde for 24 hours at room temperature (RT). Automated dehydration of tissues and subsequent paraffin embedding was performed with a Leica ASP 300S (Leica) device. Paraffin blocks, prepared with a Tissue-Tek device (Sakura), were cut into 5-μm sections. For LMMPs, the LMMP was isolated from fixed tissue or fresh tissue under a dissection microscope by peeling off the outer muscle layer of the GI tract. The tissue was fixed for 15–80 minutes in 4% paraformaldehyde at RT.

### Histopathology

LMMPs from the small were stained for nicotine amide dinucleotide phosphate diaphorase (NADPH-d) as previously described.^50^ NADPH-d was used to visualize nitrergic neurons in LMMPs, briefly: LMMPs were washed 2 times for 1 hour in phosphate-buffered saline (PBS), the samples were reacted for 1 hour at +37°C in PBS containing 0.3 % Triton-X-100 with 0.1 mg nitroblue tetrazolium chloride and 1 mg NADPH/mL added right before use, and finally washed 6 times for 5 minutes in PBS. The number of cells per image was quantified with ImageJ.

### Immunohistochemistry

Sections were deparaffinized with a xylene-alcohol-water series. Antigen retrieval was performed by boiling the samples for 10 minutes in fresh 10 mM citrate buffer (pH 6.0 + 0.05% Tween 20) and then, in the buffer, cooled to RT. Quenching of endogenous peroxidase was carried out by adding 1:53 H_2_O_2_ in TBS solution for 30 minutes at RT. After washing with TBS-T (TBS with 0.1% Tween 20), blocking (3.0% normal goat serum in TBS-T) was performed by adding for 30 minutes at RT. Incubation with the primary antibody solution (1.5% normal goat serum in TBS-T, rabbit anti ubiquitin C-terminal hydrolase L1 [PGP9.5] 1:250 BML PG9500, Enzo Life Sciences; rabbit anti neuronal class III β-Tubulin [TUJ1] 1:1000, MRB-435P, Covance) was performed overnight at +4°C. We were unable to use the same pan-neuronal marker PGP9.5 used to characterize *Gdnf*^*WT/Hyper*^ mice because this antibody is no longer produced by Enzo Life Sciences. Instead, we used antibodies against pan-neuronal marker TUJ1 to analyze the duodenum of adult *Gdnf*^*WT/cHyper*^ mice.

Incubation with the secondary antibody solution (1.5% normal goat serum in TBS-T [Biotinylated anti-rabbit 1:200 Vector kit; Goat anti-rabbit Alexa 488 1:400 Invitrogen A11034, Donkey anti-mouse Cy3 Jackson 715-165-150 1:400]) was performed for 90 minutes at RT. For biotinylated secondary antibodies, signal was enhanced with an ABC-reaction kit (PK-4001, Vector Laboratories) and visualized with a DAB-kit (SK-4100, Vector Laboratories). Samples were dehydrated in a water-alcohol-xylene series and mounted with Depex. Fluorescent whole-mount samples were mounted in glycerol and fluorescent paraffin sections were mounted in Immu-mount (Thermo-Scientific). Samples were imaged with an Olympus BX-UCB microscope or scanned with a digital slide scanner (3DHistech, Budapest, Hungary). As negative controls, either the primary or the secondary antibody was omitted.

LMMPs from the small intestine were permeabilized with 1% Triton-X100 (Thermo Fisher Scientific) in PBS for 1–2 hours. Next, the tissues were blocked in 5% normal donkey serum (Abcam) and 0.3% Triton-X100 (Thermo Fisher Scientific) in PBS for 1 hour at RT followed by incubation with a goat anti-calretinin or goat anti-NOS1 and anti-HuD antibody (goat anticalretinin 1:500 CG1 swant, goat anti NOS1 1:500 abcam [ab1376], rabbit anti ELAV-like protein 4 [HuD] 1:500 Invitrogen PA5-79199; 0.5% normal donkey serum in 0.3% Triton-X100) overnight at 4°C. Quantification of cholinergic neurons using a ChAT-antibody is difficult because of the appearance of punctuate staining.^51^ Thus, we used an antibody against calretinin that is expressed in most of the myenteric cholinergic neurons.^22^^,^^24^^,^^52^ After washing thrice in PBS (15 minutes per wash), the tissues were incubated for 1 hour at RT with donkey anti-rabbit Alexa 488 1:500 (Abcam, Ab150065) and donkey anti-goat Alexa 594 1:500 (Abcam, Ab150132) secondary antibodies (0.5% normal donkey serum in 0.3% Triton-X100). Finally, the tissues were washed thrice in PBS (15 minutes per wash), and a coverslip was placed on the positively charged slide (Thermo Fischer Scientific, Menzel-Gläser Superfrost plus) using mounting media (Thermo Fischer Scientific, Shandon Immu-Mount).

### RNA Isolation

RNA was isolated from snap frozen tissues or cells from primary cultures using RNaqueous Micro kit (Ambion, Thermo Fischer Scientific) for embryonic samples or TRIzol reagent (Invitrogen, Thermo Fischer Scientific) according to the manufacturer’s instructions. DNase I treatment was similarly performed according to the manufacturer’s instructions (Thermo Fischer Scientific). cDNA was synthesized from 50–400 ng of RNA (equal amount of RNA was used in each experiment).

### Quantitative Real-Time PCR

qPCR reactions were performed with the LightCycler 480 real-time PCR system (Roche Diagnostics, Basel, Switzerland) using LightCycler 480 SYBR Green I Master, complemented with 2.5 pmol of primers into a final volume of 10 μL on white 384-well plates sealed with an adhesive plate sealer (04729749001, Roche, Basel, Switzerland). In each reaction 2.5 μL of the diluted cDNA was used. Two or 3 replicates of each reaction were included in the qPCR runs. The following qPCR program recommended for SYBRgreen was used: preincubation 10 minutes at 95°C, amplification 10 seconds at 95°C, 15 seconds at 60°C, 15 seconds at 72°C for 45 cycles, melting curve 5 seconds at 95°C, 30 seconds at 55°C, continuous acquisition mode at 95°C with 2 acquisitions per degree Celsius, and cooling 10 seconds at 40°C. The results were analyzed with LightCycler 480 Software Release 1.5.0 SP1 using the Absolute Quantification/2nd Derivative Max calculation. Beta-actin (*bAct*) and Glyceraldehyde-3-phosphate dehydrogenase (*Gapdh*) were used as reference genes. In qPCR analysis where *Gdnf*^*cHyper*^ x Del-Cre mice were used (Figure 6*A*, *E*), both *bAct* and *Gapdh* were applied as reference genes. No difference was observed between *Gapdh* alone as a reference gene versus both *bAct* and *Gapdh* combined. Therefore, in the rest of the qPCR analysis *Gapdh* was used as a sole reference gene.

**Table:** 

*mbAct*	ctaaggccaaccgtgaaaag	accagaggcatacagggaca
*mAche*	ccctcgctgaactacaccac	taagggcttcaggttcaggc
*mCalb2*	cgaagagaatttccttttgtgc	tgtgtcatacttccgccaag
*mCart*	cgagaagaagtacggccaag	ctggcccctttcctcact
*mCasz1*	gctccatcccaaacacacac	gtagctcaggcagttggagg
*mChat*	tgtgtgagcactcccctttt	cgagcttcttgttgcctgtc
*mElavl4*	tgctcctgtttcccctca	cctgtcccttcagttcctca
*mEtv1*	cccagagattttgcatatgactc	gccttctgttctgcttgga
*mEtv3*	gcccaactacccgttcatca	gcacatcgccagtaggagaa
*mGal*	tgcctgcaaaggagaagagag	ccacctccagttgtaactccc
*mGapdh*	gcctcgtcccgtagacaaaa	atgaaggggtcgttgatggc
*mGdnf* ex2-3	cgctgaccagtgactccaatatgc	tgccgcttgtttatctggtgacc
*mGfap*	tcgagatcgccacctacag	gtctgtacaggaatggtgatgc
*mGfra1*	ttcccacacacgttttacca	gcccgatacattggatttca
*mIl1b*	agttgacggaccccaaaag	agctggatgctctcatcagg
*mIl1ra*	aaccagctcattgctgggtactta	gcccaagaacacactatgaaggtc
*mIl23*	gctgtgcctaggagtagcag	tggctgttgtccttgagtcc
*mNos1*	ctcgggcataccctcacttc	atgttgacgtcatcccccac
*mRet* t2	tcccttccacatggattga	atcggctctcgtgagtggta
*mS100b*	aacaacgagctctctcacttcc	ctccatcactttgtccacca
*mTbx3*	gcatcctctcctgctgtctc	gtgctcctccttgctctcag
*mTnfa*	gatcggtccccaaagggatg	tgagggtctgggccatagaa
*mTgfb*	tggagcaacatgtggaactc	cagcagccggttaccaag
*mTubb3*	gcgcatcagcgtatactacaa	ttccaagtccaccagaatgg
*mUchl1*	gggcagcagctctgtaagaa	ctggagggtccattgcttgt
*mVip*	gcctctctttggaccacctt	ctccttcaaacggcatcct
*mZfhx4*	gccttttcggttggtgctta	ttcatccagcctgtcaggga

### MicroRNA Conservation

Evolutionary conservation of miR seed sequences in Gdnf 3’UTR was assessed using Targetscan (http://www.targetscan.org/).

### MicroRNA Expression

Samples from E13.5 WT mice were analyzed of microRNA expression. TaqMan MicroRNA Assay reactions kit (Applied Biosystems/Thermo Fisher Scientific) was used according to the manufacturer’s recommendations with minor modifications.

MicroRNAs miR9, miR30a, miR33a, miR96, miR129, miR133b, and miR204 were selected for analysis based on previous analyses showing predicted putative conserved binding sites in the *Gdnf* native 3’UTR.^14^ Briefly, RNA was isolated using RNaqueous Micro kit (Ambion, Thermo Fischer Scientific) or TRIzol reagent (Invitrogen, Thermo Fischer Scientific) as described previously. cDNA was synthesized with TaqMan MicroRNA Reverse Transcription Kit (Applied Biosystems/Thermo Fisher Scientific) using Megaplex RT Primers (Applied Biosystems/Thermo Fisher Scientific) without preamplification as recommended by the manufacturer. For real-time PCR reactions 10–12.5 ng of the cDNA was used and each sample was run in triplicate. In the pilot experiment, microRNA expression was normalized to snoRNA202 and snoRNA234 and geometric mean was taken. No difference was observed between snoRNA202 alone as a reference gene versus both snoRNA202 and snoRNA234 combined. Therefore, in subsequent analysis snoRNA202 was used as a sole reference gene.

### Enteric Primary Culture

Small intestines from E12.5 mouse embryos were collected (20–30 per isolation) in Hank's Balanced Salt Solution (Thermo-Scientific, 14175-053). Small intestines were then digested in 5 mL of medium (Dulbecco's modified Eagle's medium–F12, 100 μg/mL normycin) that contained 0.2% Trypsin at 37°C for 20 minutes. Ten milliliter of medium containing 10% fetal bovine serum was added after which tissues were treated with 0.01% DNAse I (Sigma) for 10 minutes at 37°C. Homogenization of the digested tissues was done using a 300-μL pipette. The cell suspension was centrifuged at 107 *g* for 10 minutes and cells were then recovered in 10% fetal bovine serum. The cells were seeded in gelatin-coated 24-well plates at a 100,000–150,000 cells/well density and cultured in an incubator under the conditions of 5% CO_2_/95% at 37°C for 24 hours. The next day, the medium was replaced with a serum-free medium containing 1% N-2 supplement (Thermo Fischer Scientific, 17502048), and the cells were grown for 24 hours (DIV1). Then, GDNF (10 ng/mL) or vehicle was added for 24 hours (DIV2), cells were finally collected (DIV3) and RNA was extracted.

### siRNA Transfection

At DIV1 when the medium was replaced with serum-free medium containing 1% N-2 supplement, the cells were transfected using 2.5 μL of lipid-based DharmaFECT 3 Transfection Reagent along with control (mock) siRNA or Etv1-, Etv3-, or Tbx3-specific siRNAs (5 pmol/well; horizondiscovery and cultured for 24 hours. Lipid-based DharmaFECT 3 Transfection Reagent mostly transfects neuronal progenitors cells and not neurons.^53^ Different combinations of Etv1-, Etv3-, or Tbx3-specific siRNAs were also tested. This was followed by incubation with GDNF for the last 24 hours as indicated. Thus briefly, the experiments were performed under the following culture conditions: 24 hours 10% fetal bovine serum, 24 hours N2 (combined with mock siRNA or specific siRNAs), and then GDNF for the last 24 hours followed by analysis. Transfection was performed according to manufacturer’s recommendations.

### Gastrointestinal Transit Time and Stool Collection

Before the transit time experiment, adult mice were placed into separate clean cages for 24-hour stool collection (dry stool size). Then, mice were fed a 100-μL bolus of 5% medicinal carbon (Leiras) in 10% sucrose solution at 3, 12, and 18 months of age. Stool pellets were collected until the first stools containing black carbon were noticed.

When collecting samples for stool water content analysis, adult mice were placed individually in separate clean cages without bedding for 20 minutes. The stool samples were collected directly in sealed Eppendorf tubes. After initial weighing, the samples were left to air dry in the fume hood for 1 week.

### High-Performance Liquid Chromatography

For high-performance liquid chromatography, snap frozen tissue samples were homogenized by sonication (10 x 3 seconds) and centrifuged at +4°C, at 20,800 *g* for 35 minutes. A 300 μL of sample was centrifuged in Vivaspin filter tubes at +4°C, 9000 rpm for 35 minutes. Neurotransmitter levels and metabolites were measured by high-performance liquid chromatography as previously described.^54^

### Ussing Chamber—Short-Circuit Current (Isc) and TER Measurements

Following euthanasia, the colon was removed, opened along the mesenteric border, and mounted onto 0.031 cm^2^ sliders before inserting the sliders into an Ussing chamber system with a voltage-clamp apparatus (EasyMount, Physiological Instruments, San Diego, CA). Each side of the Ussing chamber was immediately filled with a physiological buffer solution and continuous oxygen flow to the chamber was applied. After a 10-minute equilibration period, short-circuit current (Isc) and TER were recorded under voltage-clamp conditions. The Isc was generated with reference to Ag/AgCl electrodes connected to the chamber halves via 4% agar–3 M KCl salt bridges. TER was obtained by pulsing a 5-mV step change in voltage clamp mode and measuring the resulting change in Isc. All electrophysiological values were normalized to the tissue area (0.031 cm^2^) and recorded with Acquire and Analyze 2.3. software (Physiological Instruments).

### Experimental Design and Statistical Analysis

All values are presented as mean ± standard error of the mean. Statistical significance level was set at *P* < .05. Statistical analysis was performed with GraphPad Prism 7.04 software. Appropriate statistical tests were used for each dataset: 1- or 2-way analysis of variance followed by Tukey honest significant difference or unpaired 2-tailed *t* test. To test for normality of the data the Kolmogorov-Smirnov test and the Shapiro-Wilk tests were used. Animal cohort sizes were selected keeping in mind the 3 R’s (replacement, reduction and refinement) principles of animal welfare. The researcher was kept blind to the genotypes of quantified samples always when it was technically possible. The similarity of variances between each data set was tested using the F test.
